# Exploiting genetic diversity and gene synthesis to identify superior nitrogenase NifH protein variants to engineer N_2_-fixation in plants

**DOI:** 10.1038/s42003-020-01536-6

**Published:** 2021-01-04

**Authors:** Xi Jiang, Lucía Payá-Tormo, Diana Coroian, Inés García-Rubio, Rocío Castellanos-Rueda, Álvaro Eseverri, Gema López-Torrejón, Stefan Burén, Luis Manuel Rubio

**Affiliations:** 1 Centro de Biotecnología y Genómica de Plantas, Universidad Politécnica de Madrid, Instituto Nacional de Investigación y Tecnología Agraria y Alimentaria, Pozuelo de Alarcón, 28223 Madrid, Spain; 2 Departamento de Biotecnología-Biología Vegetal, Escuela Técnica Superior de Ingeniería Agronómica, Alimentaría y de Biosistemas, Universidad Politécnica de Madrid, 28040 Madrid, Spain; 3 Centro Universitario de la Defensa, Ctra. de Huesca s/n, 50090 Zaragoza, Spain; 4 Department of Biosystems Science and Engineering, ETH Zürich, 4058 Basel, Switzerland

**Keywords:** Molecular engineering in plants, Iron

## Abstract

Engineering nitrogen fixation in eukaryotes requires high expression of functional nitrogenase structural proteins, a goal that has not yet been achieved. Here we build a knowledge-based library containing 32 nitrogenase *nifH* sequences from prokaryotes of diverse ecological niches and metabolic features and combine with rapid screening in tobacco to identify superior NifH variants for plant mitochondria expression. Three NifH variants outperform in tobacco mitochondria and are further tested in yeast. *Hydrogenobacter thermophilus* (Aquificae) NifH is isolated in large quantities from yeast mitochondria and fulfills NifH protein requirements for efficient N_2_ fixation, including electron transfer for substrate reduction, P-cluster maturation, and FeMo-co biosynthesis. *H. thermophilus* NifH expressed in tobacco leaves shows lower nitrogenase activity than that from yeast. However, transfer of [Fe_4_S_4_] clusters from NifU to NifH in vitro increases 10-fold the activity of the tobacco-isolated NifH, revealing that plant mitochondria [Fe-S] cluster availability constitutes a bottleneck to engineer plant nitrogenases.

## Abstract (web summary)

Jiang et al. show that nitrogenase NifH component of *Hydrogenobacter thermophilus* fulfils the quantity and functional requirements to engineer efficient N_2_ fixation in mitochondria. This study contributes towards future efforts engineering diazotrophs in eukaryotic cells.

## Introduction

Nitrogen (N) fertilizers used to increase crop productivity in intensive agriculture practices pollute groundwater and release greenhouse gasses^1^. On the other hand, subsistence agriculture practices including poor N fertilization produce low and inconsistent yields causing malnutrition and poverty^2,3^. There is large interest in engineering cereal crop varieties capable of acquiring their own N^4^. One approach to this outcome relies on functional expression of a nitrogenase enzyme by the cereal plant^5^. Nitrogenases are prokaryotic, O_2_-sensitive, two-component metalloproteins that convert inert N_2_ into biologically useful NH_3_^6–8^. The most efficient and widespread variant, the molybdenum nitrogenase, is composed of an Fe protein (*nifH*-encoded) and a MoFe protein (encoded by *nifD* and *nifK*). The Fe protein (NifH) donates electrons to the MoFe protein (NifDK) that in turn reduces N_2_. Nascent NifH and NifDK polypeptides need to acquire proper quaternary structure and to receive metal clusters, one [Fe_4_S_4_] cluster per NifH homodimer and two pairs of P-cluster and FeMo-co per NifDK heterotetramer, for functionality. We have recently reviewed the mechanisms and genetic requirements to assemble these cofactors and to mature NifH and NifDK into active Mo nitrogenase^9^. The large number of nitrogen fixation (*nif*) genes involved, and the sensitivity of most of the protein products towards O_2_, makes nitrogenase engineering a daunting task with issues that need to be solved stepwise.

To date, functional NifH, NifU, and NifB have been purified from mitochondria of aerobically cultured *Saccharomyces cerevisiae* cells^10,11^, while active NifU and NifH were isolated from chloroplasts of *Nicotiana benthamiana* at the end of the dark period^12^. Also, the reported low stability of the NifD protein^13^ has now been improved in two recent studies that identified key residues in the NifD sequence as susceptible to cleavage upon mitochondria import^14,15^. Notwithstanding these achievements, detailed analysis of yeast mitochondria-targeted *Azotobacter vinelandii* NifH has been hampered by low protein solubility resulting in suboptimal yields^10^. Accumulation of mostly insoluble NifH was also reported when *Klebsiella oxytoca* NifH was targeted to the tobacco mitochondria^16^. The difficulty of expressing high levels of soluble and functional NifH in yeast and tobacco poses a major problem for eukaryotic nitrogenase engineering as it is the most abundant Nif protein during N_2_ fixation^17^. The problem is exacerbated because, in addition to serving NifDK with electrons for substrate reduction, NifH is required to mature P-clusters onto NifDK and for the final steps of FeMo-co biosynthesis in complex with NifEN^9^. For these reasons it is essential to identify a NifH variant that is highly soluble and stable when expressed at very high levels in a plant cell, and that can perform all three NifH-dependent activities. One approach to achieve this outcome would be protein engineering of well-studied NifH from model diazotrophs (e.g., *A. vinelandii* or *K. oxytoca*) aimed to introduce sequences that improve stability in the mitochondria^18^. Protein engineering has been extensively employed to obtain glyphosate resistance^19^, another important trait for crops. Alternatively, mining of phylogenetically diverse *nifH* sources can be undertaken in order to find natural NifH proteins with superior properties, a strategy that was successful for NifB^11^ and for increasing carotenoid levels in “Golden Rice”^20^.

Here, 32 distinct *nifH* genes were screened for expression level and solubility in mitochondria of *N. benthamiana*. The *nifM*, *nifU*, and *nifS* genes were co-expressed because their protein products are involved in NifH folding and in the biosynthesis and delivery of its [Fe_4_S_4_] cluster^9^. The *Hydrogenobacter thermophilus* NifH was identified as vastly superior to the *A. vinelandii* NifH in terms of expression levels, solubility, and functionality both in tobacco and yeast mitochondria. Mitochondria-targeted *H. thermophilus* NifH satisfied all functional and spectroscopic requirements of a nitrogenase Fe protein when purified from yeast. The screening also pinpointed the plant mitochondria [Fe-S] cluster assembly as a bottleneck for further engineering.

## Results

### Library design and strategy for expression of mitochondria-targeted NifH in *N. benthamiana*

**Fig. 1:**
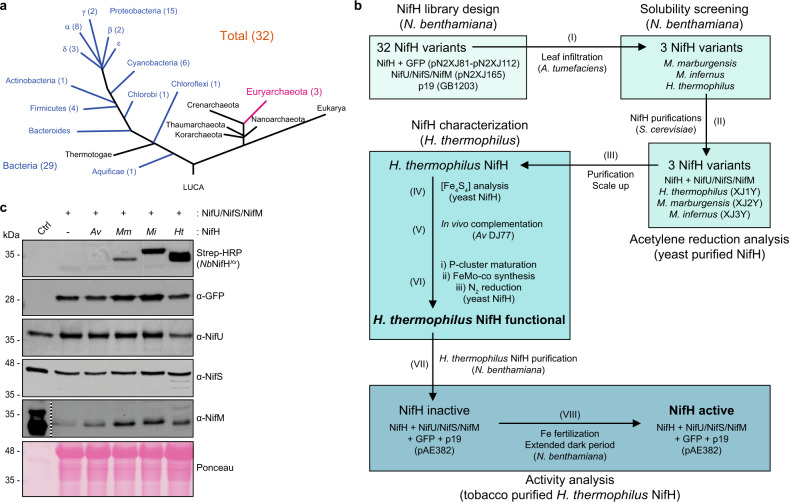
NifH library design and screening. **a** Phylogenetic tree indicating the distribution and number of the tested NifH variants among bacterial phyla (blue) and archaeal lineage (magenta). **b** Experimental workflow of this study. **c** Immunoblots of *N. benthamiana* soluble protein extracts developed with antibodies against TS, GFP, NifU, NifS, and NifM (step I of panel **b**). GFP detection was used as control to normalize effectiveness of infiltration experiments. Ponceau staining panel is shown as loading and membrane transfer control. Dotted line indicates different exposures of the same membrane. Cell-free extracts of *A. vinelandii* DJ (for NifU and NifS) or *E. coli* Rosetta (DE3) overexpressing NifM^*Av*^ (for NifM, as NifM expression levels in *A. vinelandii* is low) were used as size controls. Uncropped immunoblots are shown in Supplementary Fig. 8.

A library of 32 *nifH* sequences from phylogenetically diverse prokaryotes was designed considering one or several of the following criteria: (*i*) *nifH* genes found in confirmed diazotrophs; (*ii*) *nifH* genes from phototrophs or plant-associated bacteria; (*iii*) *nifH* genes from aerobic organisms; (*iv*) growth temperature of the *nifH* host; (*v*) *nifH* genes from archaeal representatives (Supplementary Data 1). Organized by phyla, the selection included genes from 1 Aquificae, 4 Firmicutes, 1 Actinobacteria, 15 Proteobacteria, 6 Cyanobacteria, 1 Chlorobi, 1 Chloroflexi, and 3 Euryarchaeota (Fig. 1a).

The workflow of this study is described in Fig. 1b. The gene sequences encoding the 32 NifH variants were cloned into plant vectors for *Agrobacterium tumefaciens* infiltration-mediated NifH expression in *N. benthamiana* leaves (Supplementary Table 1, see Methods section for details). The *nifH* sequences were codon-optimized for *S. cerevisiae* because codon-usage is similar to tobacco^21^ and the workflow included downstream expression of tobacco-selected NifH variants in yeast for biochemical characterization. The genes were under control of the strong and constitutive E35S promoter. Amino-terminal COX4-TS extensions were added to NifH proteins. COX4 is the 29 amino acid transit peptide of the *S. cerevisiae* mitochondria protein cytochrome c oxidase subunit IV (MLSLRQSIRFFKPATRTLCSSRYLLQQKP), whereas TS denotes the 28 amino acid Twin-Strep-Tag peptide (WSHPQFEKGGGSGGGSGGSAWSHPQFEK)^22^. COX4 targeted NifH proteins to the mitochondria matrix and TS was used to enable variant-independent immunoblot detection of NifH and to facilitate its purification. Importantly, the TS-tag has been shown to not significantly affect NifH functionality^12^. COX4-TS-NifH variants are hereafter denoted as *Nb*NifH^*Xx*^ where *Nb* stands for the host *N. benthamiana*, ^*Xx*^ denotes variants collectively, and other superscripts indicate the species from which NifH sequence was obtained. Vectors with *Nb*NifH^*Xx*^ constructs additionally contained a transcriptional unit for expression of the green fluorescent protein (GFP) that was used as indicator of successful leaf infiltration (Supplementary Table 1).

An auxiliary vector was constructed to co-express *A. vinelandii nifM*, *nifU,* and *nifS* and target their protein products to mitochondria via N-terminal SU9 extensions. Similar to COX4, the mitochondrial presequence of subunit 9 of the *Neurospora crassa* F_0_-ATPase^23^ (SU9) has been shown to deliver Nif proteins to *N. benthamiana* mitochondria^24^. NifU and NifS assemble [Fe-S] clusters destined for Nif proteins in *A. vinelandii*^25^. While not essential for expression of functional NifH^*Av*^ in *S. cerevisiae* mitochondria^10^ they were required to generate high amounts of active NifB in yeast^11^. As we aimed to identify NifH variants accumulating at higher levels than NifH^*Av*^, NifU^*Av*^ and NifS^*Av*^ were included in this study. In *A. vinelandii* and other well-studied diazotrophs NifM is involved in NifH folding or dimerization prior [Fe_4_S_4_] cluster acquisition^9,26^. Despite *nifM* not being present in organisms of some selected *nifH* variants (Supplementary Data 1), this gene was always included in infiltration experiments for consistency.

### Identification of NifH proteins suitable for expression in *N. benthamiana*

*N. benthamiana* leaves were co-infiltrated with a 1:1:1 mixture of three distinct *A. tumefaciens* cultures for expression of, respectively, one *Nb*NifH^*Xx*^ variant plus GFP, the auxiliary proteins *Nb*NifM^*Av*^, *Nb*NifU^*Av*^, and *Nb*NifS^*Av*^, and the RNA silencing suppressor p19 to enhance the *nif* transgene expression (Fig. 1b)^27^. Protein extracts were prepared from the *N. benthamiana* leaves three days after infiltration and analyzed for accumulation of soluble *Nb*NifH^*Xx*^ using antibodies recognizing the TS-tag. Only two NifH variants were consistently detected among experiments (Fig. 1c, Supplementary Fig. 1a), namely those originating from *Methanocaldococcus infernus* (*Nb*NifH^*Mi*^) and *Hydrogenobacter thermophilus* (*Nb*NifH^*Ht*^). A third NifH variant from *Methanothermobacter marburgensis* (*Nb*NifH^*Mm*^) was detected at low levels at one occasion. In contrast, analysis of total extracts prepared from the infiltrated tobacco leaves showed that, although accumulation levels of the *Nb*NifH^*Xx*^ proteins varied significantly, 25 of the 32 variants could be detected (Supplementary Fig. 1b). Only *Nb*NifH expression of variants from *Bradyrhizobium japonicum*, *Rhizobium leguminosarum* bv. *trifolii*, *Herbaspirillum seropedicae*, *Gloeothece* sp. KO68DGA, *Rhodopseudomonas palustris*, *Methanothermobacter thermautotrophicus*, and *Frankia* sp. (strain FaC1) could not be demonstrated. Sequence alignments and 3D-modeling of NifH^*Mi*^, NifH^*Ht*^, and NifH^*Mm*^ are shown in Supplementary Fig. 2. The 3D-models did not reveal any specific feature that would explain their superior accumulation as soluble protein in tobacco mitochondria, but all three proteins originate from thermophilic organisms (Supplementary Data 1) which could possibly explain their stability and solubility.

### Activity of NifH variants isolated from mitochondria of aerobically cultured *S. cerevisiae*

*N. benthamiana* screening-identified variants and NifH^*Av*^ were expressed in *S. cerevisiae* and purified by Strep-tag affinity chromatography (STAC) to evaluate functionality when targeted to mitochondria. For this, genes encoding COX4-TS-NifH constructs were transferred to expression vectors together with *su9-nifM*^*Av*^, *su9-nifU*^*Av*^, and *su9-nifS*^*Av*^ under the control of galactose-inducible GAL1 or GAL10 promoters (Supplementary Table 2, Supplementary Fig. 3a). These COX4-TS-NifH variants expressed in aerobic *S. cerevisiae* cultures are hereafter denoted *Sc*NifH^*Mm*^, *Sc*NifH^*Mi*^, *Sc*NifH^*Ht*^, and *Sc*NifH^*Av*^ (*Sc*NifH^*Xx*^ collectively).

**Fig. 2:**
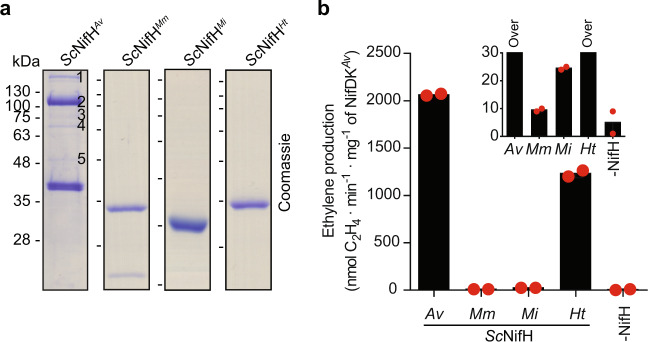
Activity of selected NifH variants purified from mitochondria of aerobically cultured *S. cerevisiae* engineered strains. **a** STAC-purified *Sc*NifH^*Av*^ (strain XJ4Y), *Sc*NifH^*Mm*^ (strain XJ2Y), *Sc*NifH^*Mi*^ (strain XJ3Y), and *Sc*NifH^*Ht*^ (strain XJ1Y) proteins. The low solubility of *Sc*NifH^*Av*^ promoted binding of contaminating proteins (as column was not saturated with *Sc*NifH^*Av*^) that were identified by peptide mass fingerprinting as: 1) Acc1p (N1P4Q3), 2 and 3) pyruvate carboxylase (N1P377), and 4) HSP70 (D2J4C2). No identification was possible for band number 5. Uncropped gels are shown in Supplementary Fig. 9. **b** ARA of STAC-purified *Sc*NifH^*Xx*^ variants. Activity using NifH^*Av*^ and NifDK^*Av*^ (positive control) was 2406 ± 53 units (nmol ethylene formed per min and mg of NifDK^*Av*^). Data represent mean values (*n* = 2 technical replicates).

While *Sc*NifH^*Mm*^, *Sc*NifH^*Mi*^, and *Sc*NifH^*Ht*^ were purified to near homogeneity (Fig. 2a), SDS-PAGE analysis of *Sc*NifH^*Av*^ showed additional slower migrating co-eluting proteins. Mass spectrometry confirmed that these were contaminants (Fig. 2a). *Sc*NifH^*Av*^ solubility was low and much protein was lost to the pellet fraction when preparing the soluble cell-free extract (CFE) explaining its poor purification yield (about 11 mg per kg of *S. cerevisiae* cells) (Supplementary Fig. 3b–e, Supplementary Table 3). The yield of *Sc*NifH^*Mm*^ was also relatively low, in line with the inferior result in the *N. benthamiana* screening. In contrast, the yields of *Sc*NifH^*Mi*^ and *Sc*NifH^*Ht*^ were ca. 20 times higher. Iron (Fe) quantification of purified samples was variable but indicated that *Sc*NifH^*Ht*^ was isolated largely as holo-protein containing one [Fe_4_S_4_] cluster per dimer (Supplementary Table 3). Consistently, immunoblot analysis showed that *Sc*NifH^*Ht*^, *Sc*NifM^*Av*^, *Sc*NifU^*Av*^, and *Sc*NifS^*Av*^ had been efficiently targeted to the mitochondria (Supplementary Fig. 4).

Activities of purified *Sc*NifH^*Xx*^ variants were determined in vitro using the acetylene reduction assay (ARA) and compared to that of NifH purified from *A. vinelandii* (denoted NifH^*Av*^). In all cases NifDK purified from *A. vinelandii* (denoted NifDK^*Av*^) was used as MoFe protein component. *Sc*NifH^*Av*^ activity was 85% of NifH^*Av*^ (Fig. 2b), supporting previous observations that STAC is suitable for purification of metal-cluster containing Nif proteins expressed in yeast^11,28^. *Sc*NifH^*Ht*^ specific activity was about half of *Sc*NifH^*Av*^, while *Sc*NifH^*Mm*^ and *Sc*NifH^*Mi*^ showed very low activities (Fig. 2b). The assay did not determine whether lower activities were due to NifH variant defects, or to incompatibility with *Sc*NifUS^*Av*^ in vivo (resulting in apo-NifH protein with low [Fe_4_S_4_] cluster occupancy) or NifDK^*Av*^ in vitro (resulting in poor electron donation). Reconstitution of *Sc*NifH^*Mm*^ [Fe_4_S_4_] clusters in vitro by either mixing with Fe, L-cysteine, DTT, and *Ec*NifS^*Av*^ (direct reconstitution) or by incubating with [Fe_4_S_4_] cluster-loaded *Ec*NifU^*Av*^ (NifU-mediated reconstitution) did not activate the protein (Supplementary Fig. 5), indicating that this NifH variant is not compatible with NifDK^*Av*^. In contrast, *Sc*NifH^*Mi*^ was activated to some extent by NifU^*Av*^, and further by direct reconstitution, indicating that the *A. vinelandii* NifUS machinery is not optimal for NifH^*Mi*^ (Supplementary Fig. 5). However, activities were very low compared to the as-isolated *Sc*NifH^*Ht*^ protein (Fig. 2b). This could be explained by NifH^*Ht*^ harboring more of the conserved amino acid residues known to be important for the interaction with NifDK^*Av*^ (Supplementary Fig. 2).

Importantly, soluble accumulation of *Sc*NifH^*Ht*^ in mitochondria was 20-fold higher than *Sc*NifH^*Av*^ (Supplementary Table 3), which translates into at least 10-fold higher in vivo activity and fulfills NifH quantity requirements for nitrogenase engineering. Thus, *Sc*NifH^*Ht*^ was further characterized.

### *Sc*NifH^*Ht*^ exhibits NifH-characteristic spectroscopic signals and is functional in vivo

**Fig. 3:**
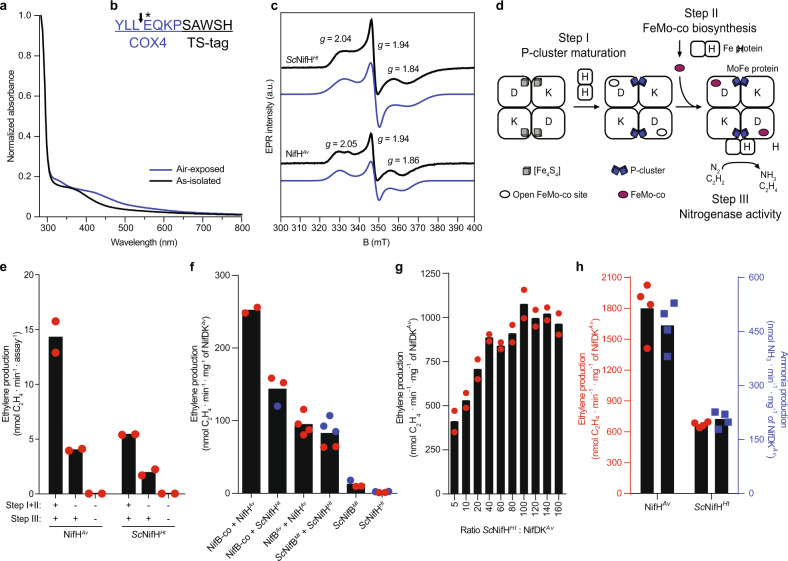
Characterization of *Sc*NifH^*Ht*^. **a** UV–vis absorption spectra of as-isolated and air-exposed *Sc*NifH^*Ht*^. **b** Processing site (black arrow) of COX4 mitochondria targeting signal (blue) as determined by N-terminal sequencing of *Sc*NifH^*Ht*^. The conversion of Q to E (marked by *) could be due to deamination processes. **c** EPR signal of as-isolated *Sc*NifH^*Ht*^ (190 μM) compared to NifH^*Av*^ (71.2 μM). Experimental data (black lines) and simulations (blue lines) of each protein are shown together with *g* values. **d** Schematic representation of NifH-dependent activities tested in **e**–**h**. Figure adapted from Burén et al.^9^. Copyright 2020 ACS under CC BY 4.0 http://creativecommons.org/licenses/by/4.0. **e** In vitro P-cluster maturation of apo-NifDK^*Av*^ present in CFE of *A. vinelandii* DJ77 (Δ*nifH*) after addition of *Sc*NifH^*Ht*^ or NifH^*Av*^ as indicated (±). Tetrathiomolybdate was added (following step I + II) to inhibit further apo-NifDK^*Av*^ activation during the ARA (step III). Data represent mean values (*n* = 2 technical replicates). **f** NifB-co and NifB-dependent in vitro FeMo-co synthesis using *Sc*NifH^*Ht*^ or NifH^*Av*^. Tetrathiomolybdate was added (following step II) to inhibit further apo-NifDK^*Av*^ activation during the ARA (step III). A 20:1 molar ratio of *Sc*NifH^*Ht*^ to NifDK^*Av*^ was used in the ARA (step III). Data represent mean values (*n* = 2 technical replicates (NifB-co + NifH^*Av*^), *n* = 3 technical replicates (NifB-co + *Sc*NifH^*Ht*^), *n* = 4 technical replicates (NifB^*Av*^ + NifH^*Av*^), *n* = 5 technical replicates (*Sc*NifB^*Mt*^ + *Sc*NifH^*Ht*^), *n* = 3 technical replicates (*Sc*NifB^*Mt*^), *n* = 4 technical replicates (*Sc*NifH^*Ht*^)). Blue and red dots correspond to independent experiments. **g** Titration of NifDK^*Av*^ activity with *Sc*NifH^*Ht*^. Positive control reactions performed with NifH^*Av*^ and NifDK^*Av*^ at 40:1 molar ratio gave 1692 ± 4 units (nmol ethylene formed per min and mg of NifDK^*Av*^). Reactions lacking NifH (negative control) gave 2.5 ± 0.8 units. Data represent mean values (*n* = 2 technical replicates). **h** ARA (red dots, left *y*-axis) and N_2_-reduction assay (blue squares, right *y*-axis) using *Sc*NifH^*Ht*^ and NifDK^*Av*^ (step III). NifH^*Av*^ was used as control. Data represent mean values (*n* = 4 technical replicates).

Purified *Sc*NifH^*Ht*^ protein presented ultraviolet–visible (UV–vis) absorption spectra typical of O_2_-sensitive [Fe-S] cluster-containing proteins (Fig. 3a). Amino-terminal sequencing revealed that amino acid residues EQKP remained after COX4 processing (Fig. 3b), where conversion of glutamine (Q) to glutamic acid (E) could be due to deamination performed by the mitochondrial matrix N-terminal amidase NTA1^29^. Electron paramagnetic resonance (EPR) confirmed that *Sc*NifH^*Ht*^ protein contained an [Fe_4_S_4_] cluster with similar signal intensity and *g*-values as NifH^*Av*^ (Fig. 3c), suggestive of successful maturation into functional Fe protein.

The NifH variant chosen to engineer N_2_-fixing plants must perform P-cluster maturation and FeMo-co biosynthesis in addition to serve as electron donor for substrate reduction. We therefore tested whether *H. thermophilus* NifH could revert the Nif^−^ phenotype of *A. vinelandii* DJ77 (Δ*nifH* strain)^30^. For this, *ts*-*nifH*^*Ht*^ was introduced by transformation into DJ77 and the resulting strain UW481 was tested for diazotrophic growth and in vivo acetylene reduction activity. UW481 showed diazotrophic growth both in solid and liquid media (Supplementary Fig. 6a, b), and immunoblot analysis demonstrated sustained *Av*NifH^*Ht*^ expression and acetylene reducing activity indicative of active nitrogenase (Supplementary Fig. 6c, d). These data strongly indicate that NifH^*Ht*^ can replace the functions of native *A. vinelandii* NifH to some extent, which requires productive interactions with at least apo-NifDK^*Av*^, NifDK^*Av*^, and NifEN^*Av*^ proteins.

### *Sc*NifH^*Ht*^ is active in substrate reduction, P-cluster formation and FeMo-co synthesis

Each individual NifH-dependent activity was then analyzed in vitro using pure *Sc*NifH^*Ht*^ preparations (Fig. 3d). P-cluster maturation was determined by supplementing CFE of *A. vinelandii* DJ77 (Δ*nifH*) with *Sc*NifH^*Ht*^. The DJ77 extract is devoid of FeMo-co and contains inactive apo-NifDK^*Av*^ with immature P-clusters. The P-cluster maturation assay using DJ77 CFE relies on positive outcomes of three distinct activities performed in two sequential reactions (Fig. 3d). In the first reaction (Step I + II) pure NifH and FeMo-co are added to DJ77 CFE resulting in NifH-dependent reductive coupling of the two [Fe_4_S_4_] P-cluster precursors to form mature P-clusters (Step I), followed by FeMo-co insertion into P-cluster containing apo-NifDK^*Av*^ to generate active NifDK^*Av*^ (Step II) (Fig. 3d). Tetrathiomolybdate is then added to prevent further FeMo-co insertion, separating the maturation (Step I + II) and activity (Step III) reactions. Activation of DJ77 apo-NifDK^*Av*^ by *Sc*NifH^*Ht*^ demonstrated its P-cluster maturation activity (Fig. 3e).

In vitro FeMo-co synthesis (Fig. 3d, Step II)^9^ was determined by combining purified preparations of *Sc*NifH^*Ht*^, apo-NifDK^*Av*^ containing P-clusters but devoid of FeMo-co^31^, apo-NifEN^*Av*^ containing permanent [Fe_4_S_4_] clusters but lacking FeMo-co precursor^32^, Mo, homocitrate, and either the FeMo-co precursor NifB-co bound to the carrier protein NifX^*Av*^^33^ or NifB protein supplemented with Fe and S^34^. As for the P-cluster maturation assay, tetrathiomolybdate was added before the ARA (Fig. 3d, Step III). Figure 3f shows that *Sc*NifH^*Ht*^ supported FeMo-co synthesis in vitro. Importantly, *Sc*NifH^*Ht*^ and *Sc*NifB^*Mt*^ (*Methanothermobacter thermautotrophicus* NifB isolated from *S. cerevisiae*)^11^ acted together in the NifB-dependent in vitro FeMo-co synthesis assay in which NifB-co was concomitantly synthesized by *Sc*NifB^*Mt*^ rather than added in purified form. This result proved compatibility of two essential proteins for N_2_ fixation, *Sc*NifH^*Ht*^ and *Sc*NifB^*Mt*^, when produced in yeast mitochondria. It also showed interspecies compatibility with NifDK^*Av*^ and NifEN^*Av*^, altogether constituting the conserved biochemical core of nitrogenase.

*Sc*NifH^*Ht*^ activity in substrate reduction was demonstrated by the ARA and by reduction of N_2_ into NH_3_. ARA titration was carried out with a fixed quantity of NifDK^*Av*^ and increasing amounts of *Sc*NifH^*Ht*^. Maximum NifDK^*Av*^ activity was achieved at molar *Sc*NifH^*Ht*^ to NifDK^*Av*^ ratios larger than 40 (Fig. 3g), similar to reactions with the natural counterpart NifH^*Av*^ ^35^. This result suggests that the maximum activity that can be achieved combining *Sc*NifH^*Ht*^ with NifDK^*Av*^ is 1000 units (i.e., half of the activity with NifH^*Av*^). In addition, *Sc*NifH^*Ht*^ supported N_2_ reduction into NH_3_ by NifDK^*Av*^. Importantly, the ratio of NH_3_ to ethylene produced by NifDK^*Av*^ was similar independently of using NifH^*Av*^ or *Sc*NifH^*Ht*^ (Fig. 3h).

### As-isolated *Nb*NifH^*Ht*^ was inactive but could be activated by [Fe_4_S_4_] cluster reconstitution

**Fig. 4:**
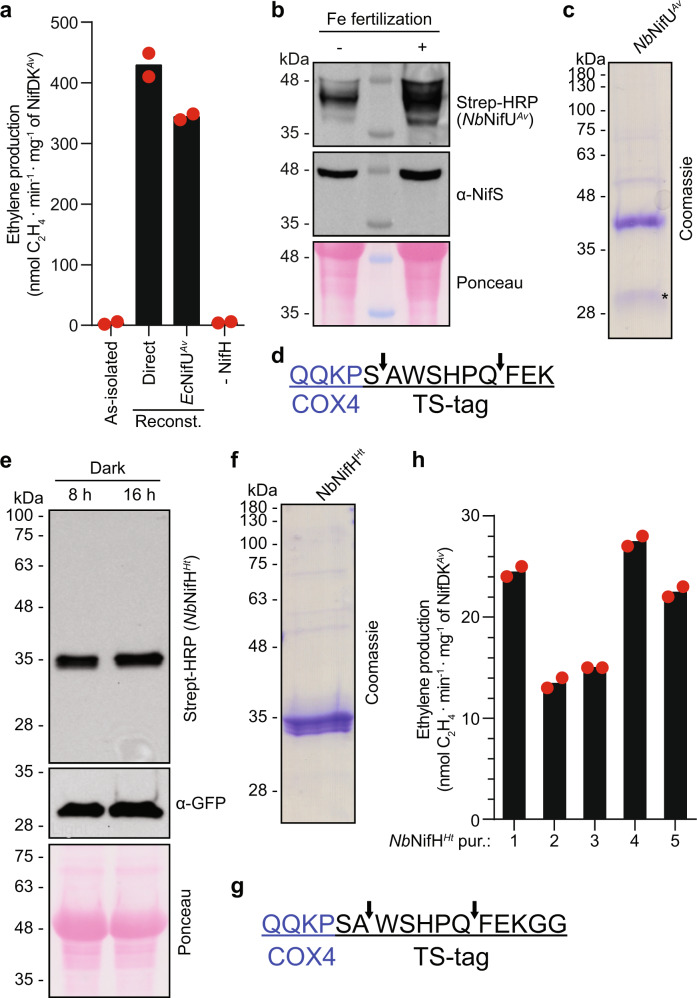
Characterization of the *Nb*NifH^*Ht*^ protein. **a** Activation of as-isolated *Nb*NifH^*Ht*^ protein with [Fe_4_S_4_] clusters either by direct chemical synthesis or by *Ec*NifU^*Av*^-mediated reconstitution. Activity using NifH^*Av*^ (positive control) was 1773 ± 10 units (nmol ethylene formed per min and mg of NifDK^*Av*^). Data represent mean values (*n* = 2 technical replicates). **b** Immunoblots showing the effect of Fe fertilization on *Nb*NifU^*Av*^ and *Nb*NifS^*Av*^ total protein expression. NifS antibody was probed on the same membrane after incubation with Streptactin-HRP. **c** *Nb*NifU^*Av*^ protein purified from tobacco plants. The lower band (marked by *) indicates a faster migrating *Nb*NifU^*Av*^ polypeptide. **d** Processing sites (black arrows) of the COX4 mitochondria targeting signal (blue) as determined by N-terminal sequencing of the full-length and the faster migrating *Nb*NifU^*Av*^. **e** Immunoblots showing accumulation of *Nb*NifH^*Ht*^ at the end of 8 h or 16 h night (dark period). **f** *Nb*NifH^*Ht*^ protein purified from tobacco leaves. **g** Processing sites (black arrows) of COX4 signal (blue) as determined by N-terminal sequencing of *Nb*NifH^*Ht*^. **h** ARA of five independent *Nb*NifH^*Ht*^ STAC-purifications (1–5). Measured activities using NifH^*Av*^ (positive controls) and without NifH (negative controls) were, respectively, 2406 ± 53 and 4.6 ± 1.3 (purification 1), 1773 ± 10 and 3.2 ± 2.2 (purifications 2 and 3), and 1692 ± 3.9 and 2.5 ± 0.8 (purifications 4 and 5). All activities are in nmol ethylene formed per min and mg of NifDK^*Av*^. Data represent mean values (*n* = 2 technical replicates). Uncropped immunoblots and gels are shown in Supplementary Fig. 10.

*Nb*NifH^*Ht*^ was purified from *A. tumefaciens*-infiltrated leaves of *N. benthamiana*. Plants were grown under long-day conditions (16 h light/8 h dark) and leaves were processed at the end of the dark period. Genes encoding *Nb*NifH^*Ht*^, *Nb*NifM^*Av*^, *Nb*NifU^*Av*^, and *Nb*NifS^*Av*^ (together with p19 and GFP) were piled up in a single plant-expression vector for co-expression (Methods section and Supplementary Table 1). Purified *Nb*NifH^*Ht*^ did not exhibit brown color of [Fe-S] clusters and was inactive in the ARA when combined with NifDK^*Av*^ (Fig. 4a). Therefore, we reconstituted *Nb*NifH^*Ht*^ [Fe_4_S_4_] cluster in vitro either by mixing with Fe, L-cysteine, DTT, and *Ec*NifS^*Av*^ (direct reconstitution), or by incubating with [Fe_4_S_4_] cluster-loaded *Ec*NifU^*Av*^ (NifU-mediated reconstitution). Both methods activated the *Nb*NifH^*Ht*^ as determined by the ARA (Fig. 4a), demonstrating that the protein was correctly folded but lacked its [Fe_4_S_4_] cluster. This result suggested that insertion and/or stability of *Nb*NifH^*Ht*^ [Fe_4_S_4_] cluster was poor in mitochondria of leaves.

### Fe fertilization of the soil increases soluble NifU in mitochondria of *N. benthamiana*

One explanation for the low [Fe_4_S_4_] cluster content of *Nb*NifH^*Ht*^ could be insufficient Fe availability in the soil. We observed that accumulation of *Nb*NifU^*Av*^, but not of *Nb*NifS^*Av*^, increased when the water used to irrigate the *A. tumefaciens*-infiltrated plants was supplemented with Fe (Fig. 4b). Sulfur was not supplemented in soil as the infiltration solution contained Mg_2_SO_4_. Although Fe fertilization tripled the yield of STAC-isolated *Nb*NifU^*Av*^, the average Fe content of 2 Fe atoms per protein was not affected (Supplementary Fig. 7a, b, Supplementary Table 4). This could be due to the loss of transient NifU [Fe-S] clusters during purification, and it is not a surprising outcome as isolation of *Ec*NifU^*Av*^ containing only the permanent [Fe_2_S_2_] clusters has been previously observed^36^. Immunoblots detected two differently migrating *Nb*NifU^*Av*^ species in purifications from tobacco leaves (Fig. 4c). Amino-terminal sequencing showed that both species were cleaved either one or seven amino acids into the TS-tag (Fig. 4d). As both *Nb*NifU^*Av*^ species showed the same N-termini processing, we concluded that the faster migrating polypeptide was truncated at the C-terminus.

### Extended dark period combined with Fe fertilization produced active *Nb*NifH^*Ht*^ in mitochondria of *N. benthamiana* leaves

The soil of *N. benthamiana* plants expressing *Nb*NifH^*Ht*^ was fertilized with Fe to increase Fe availability. In addition, the dark period preceding leaf harvest was extended from 8 h to 16 h hypothesizing that longer darkness would lower intracellular O_2_ and stabilize *Nb*NifH^*Ht*^ [Fe_4_S_4_] cluster. Dark period extension did not increase *Nb*NifH^*Ht*^ accumulation (Fig. 4e) but allowed for isolation of active protein as shown below. About 6 mg of *Nb*NifH^*Ht*^ was consistently isolated per kg of *N. benthamiana* leaves (Fig. 4f, Supplementary Fig. 7c, Supplementary Table 5). Amino-terminal sequencing showed that mitochondria-targeted *Nb*NifH^*Ht*^ accumulated as two species (similar to *Nb*NifU^*Av*^), one in which two amino acid residues from the TS-tag were removed with the COX4 signal and another that was processed five amino acid residues further into the TS-tag (Fig. 4g).

Functionality of *Nb*NifH^*Ht*^ isolated from leaves of Fe fertilized tobacco plants following 16 h of darkness was determined using ARA. *Nb*NifH^*Ht*^ preparations consistently showed activities but these were low compared to those of [Fe_4_S_4_] cluster-reconstituted *Nb*NifH^*Ht*^ (Fig. 4h). This result suggested that *Nb*NifH^*Ht*^ accumulated as two species in tobacco mitochondria, where inactive protein likely lacking [Fe_4_S_4_] cluster was more abundant than functional and [Fe_4_S_4_] cluster-containing *Nb*NifH^*Ht*^. Consistently, the Fe content of purified *Nb*NifH^*Ht*^ preparations were below detection limit (Supplementary Table 5). Altogether the results indicate that while soluble *Nb*NifH^*Ht*^ accumulates in good quantity in mitochondria of *N. benthamiana* leaves, engineering of additional protein components or biosynthetic pathways will be required to improve [Fe_4_S_4_] cluster acquisition or stability.

## Discussion

The first study reporting production of active NifH in yeast proved that mitochondria is a suitable organelle for hosting O_2_-sensitive Nif proteins under aerobic growth conditions^10^. Despite being a valid proof-of-concept, further developments with *A. vinelandii* NifH were limited by low yields as only a small portion was soluble in the mitochondrial matrix. Similar solubility issues were later reported for *K. oxytoca* NifH targeted to *N. benthamiana* mitochondria^16^ and are confirmed in this study using immunoblot screening and STAC. Identifying the best possible NifH protein for eukaryotic (plant) expression was therefore of uttermost importance. NifH is the most abundant Nif protein required for N_2_ fixation in *A. vinelandii*^17^. Besides being the Fe protein component of Mo nitrogenase, NifH is essential to the assembly of both NifDK cofactors, namely the P-cluster and the FeMo-co^9^.

NifH proteins for nitrogenase engineering in plants should: (i) be stable and soluble at high levels in the mitochondrial matrix, and (ii) be compatible with the NifDK component from a well-studied model-diazotroph if their own NifDK components are not available in purified form. Compatibility is important when evaluating function of candidate NifH variants. In our case it meant that any selected NifH^*Xx*^ should be compatible with NifM^*Av*^ (if NifH^*Xx*^ is not NifM-independent), NifUS^*Av*^ for maturation and [Fe_4_S_4_] cluster synthesis/insertion, and NifDK^*Av*^ for nitrogenase activity measurements. We note that this requirement introduces a selection bias and that the screening could have overlooked NifH variants that were superior to that of *H. thermophilus* if combined with different NifDK.

The NifH variants tested in this study were selected from a curated dataset of hundreds of NifH sequences by favoring aerobic or plant-associated origins, to overcome the inherent O_2_-sensitivity of NifH, and functionality at moderate temperatures. We also hypothesized that NifH variants from archaea could function better in a eukaryotic environment as this domain of life is believed to be more closely related to the Eukaryota^37^, and because our previous work expressing archaeal NifB variants in yeast had shown them to be superior to those of bacterial origin^11^.

We expected that most NifH variants would be partly soluble in tobacco mitochondria when expressed together with the accessory proteins NifU^*Av*^, NifS^*Av*^, and NifM^*Av*^. However, only NifH from *M. infernus* and *H. thermophilus* were consistently detected in soluble tobacco extracts, in addition to *M. marburgensis* that was occasionally detected at lower levels. Two of these NifH proteins originated from archaea and the third from a bacterium. One possibility could be that the NifM^*Av*^ protein was not expressed at sufficient levels in the tobacco mitochondria and that only these three NifH variants did not require NifM for maturation. However, low levels of NifM expression appear to be enough for NifH maturation in *K. oxytoca*^38,39^. A more plausible explanation can be found in the thermophilic nature of *M. infernus*, *H. thermophilus,* and *M. marburgensis*. It has recently been reported that the temperature inside respiring mitochondria of cultured human cells is around 50 °C^40^, even when the external medium is maintained at 38 °C. Whether the same drastic effect on temperature holds true for mitochondria of a leaf cell is not known to us, but it could explain in part the outcome of our NifH screening. None of the two highest expressed NifH proteins originated from proven diazotrophs. We are not aware of any study investigating diazotrophy in the archaeon *M. infernus*. However, NifB^*Mi*^ cured the Nif^−^ phenotype of an *A. vinelandii nifB* mutant strain^41^ and, as NifB has no other known function than biosynthesis of nitrogenase active-site cofactors, it is likely that *M. infernus* is in fact a diazotroph. On the other hand, N_2_-fixation has been tested but not observed in *H. thermophilus* TK-6^42^. Interestingly only six NifH variants in our library originated from organisms having genes with high similarity to *A. vinelandii nifM*. Perhaps other prolyl isomerases could substitute for NifM in these organisms. Whether NifM (and the NifUS machinery) is required for maturation of the three selected NifH proteins (especially NifH^*Ht*^) in mitochondria will be investigated in future work.

Mitochondria-expressed *Sc*NifH^*Ht*^ was the only variant that supported relevant nitrogenase activity when combined with NifDK^*Av*^. Its activity corresponded to roughly half of that using *Sc*NifH^*Av*^ even if the *Sc*NifH^*Ht*^ to NifDK^*Av*^ molar ratio was increased well above 40 normally used for ARA. Emerich and Burris showed that NifH proteins can function with NifDK from other organisms^35^, but this study only combined proteins from bacteria. An optimal growth temperature of 72 °C has been reported for *H. thermophilus* TK-6^43^, which could explain lower *Sc*NifH^*Ht*^ activity in substrate reduction assays. However, our prediction from this study and previous work on NifB is that suboptimal working temperature of Nif proteins from thermophiles is a price worth paying when engineering nitrogenase in eukaryotes, as solubility and stability of these variants is so much improved.

One observation of this study was that the specific activity of as-isolated *Nb*NifH^*Ht*^ protein was lower than *Sc*NifH^*Ht*^. We think this was caused by poor [Fe_4_S_4_] cluster availability – and hence inefficient incorporation – or poor NifH [Fe_4_S_4_] cluster stability within the leaf cell mitochondria. In this context, it is not known how Fe fertilization increased accumulation of soluble *Nb*NifU^*Av*^. More available Fe could increase mitochondria [Fe-S] clusters biosynthesis and [Fe_2_S_2_] cluster occupancy in *Nb*NifU^*Av*^ which, in turn, would provide stability to the protein. A compatibility issue between *Nb*NifH^*Ht*^ and *Nb*NifU^*Av*^ and *Nb*NifS^*Av*^ is unlikely since *Ec*NifU^*Av*^ could effectively activate *Nb*NifH^*Ht*^ in vitro. *N*bNifH^*Ht*^ misfolding in mitochondria is also unlikely as it was efficiently activated by reconstitution of its [Fe_4_S_4_] cluster. It is however likely that protection by respiratory O_2_-consumption in leaf is lower than in yeast. *Nb*NifH^*Ht*^ exposure to O_2_ during leaf processing is also a possibility making this a purely technical problem. While leaves were kept in liquid nitrogen and lysis and purification were performed inside an anaerobic glove box, it is difficult to completely rule out that some O_2_ trapped within the leaf was released during tissue disruption.

In conclusion, this study shows that genetic diversity can be exploited to identify, from a very large pool of sequences, the most adequate Nif protein components to engineer a eukaryotic nitrogenase. Modular cloning techniques, gene synthesis with codon optimization, and other synthetic biology tools permit building multi-protein pathways with components of very diverse origin. In this case the NifH protein from *H. thermophilus* was identified as soluble in mitochondria of both *S. cerevisiae* and *N. benthamiana* accumulating at much higher levels than the *A. vinelandii* homologue. This example is relevant not only because the identified variant performed all three NifH-essential reactions, namely P-cluster maturation, FeMo-co biosynthesis, and NifDK^*Av*^ reduction, but also because NifH^*Ht*^ formed functional interspecies interactions with NifB, NifEN, and NifDK proteins, altogether representing the four proteins constituting the core of diazotrophy.

## Methods

### Design, assembly, and cloning of the nifH library

A curated dataset of diazotrophs^41^ was used to collect *nifH* candidates and design the library. Genes encoding *nifH* variants were codon optimized for expression in *S. cerevisiae* and the sequence encoding *pE35S::cox4*-*twinstrep* was codon optimized for expression in tobacco (Supplementary Data 1). All genetic parts were optimized using the GeneOptimizer tool (ThermoFisher) and synthesized by ThermoFisher via the Engineering Nitrogen Symbiosis for Africa (ENSA) project. The *nifH* genes were synthesized and cloned into pMA cloning vector with *BamH*I and *BstE*II restriction sites flanking each gene. The *pE35S::cox4*-*twinstrep* sequence was flanked by *Hind*III and *Bgl*II restriction sites.

pGFPGUSplus (plasmid #64401, Addgene) and the pMA vector containing *pE35S::cox4*-*twinstrep* were digested with *Hind*III and *Bgl*II and used to generate the parental vector pN2SB41, containing a pE35S::*cox4*-*twinstrep*-*gus*-tNOS transcriptional unit in which *gus* was flanked by *BamH*I and *BstE*II restriction sites. The parental vector pN2SB41 and all pMA vectors containing *nifH* variants were digested with *Bam*HI and *BstE*II and used to generate vectors pN2XJ81-pN2XJ112 (Supplementary Table 1).

pGFPGUSplus was used to generate vector pN2XJ165 containing transcriptional units for mitochondria-targeted accessory Nif proteins (*A. vinelandii* NifU, NifS, and NifM). The *su9-nifU*^*Av*^ (AAAAGGATCCAATGGCCTCCACTCGTGTCCTCG, AAAAAAGGTCACCTTAGACTTCCATTTGGGCGTGTGCG) and *su9-nifS*^*Av*^ (AAACTAGTATGGCCTCCACTCGTGTCCTCG, AAAAGAGCTCTTAACCATAGACAGGAGCAAAGGCTTTACC) genes were amplified by PCR from the yeast vector pN2GLT4^10^. Amplification reactions added flanking *BamHI* and *BstE*II (for *su9-nifU*) or *Spe*I and *Sac*I (for *su9-nifS*^*Av*^) sites. The DNA fragment containing the *su9-nifM*^*Av*^ sequence was created by overlapping PCR using primers introducing sequences homologous to those flanking the *Xho*I site of pGFPGUSplus (ATTATGGAGAAACTCGAGTTAACCATGTGCTAAGTTTTCC, TACAAATCTATCTCTCTCGAGATGGCCTCCACTCGTG, CTTTCTGAGGCCATGGAAGAGTAGGCGCGCTTCTGG, CGCGCCTACTCTTCCATGGCCTCAGAAAGATTAGCTGATG). pGFPGUSplus was first digested with *Bgl*II and *BstE*II to insert *su9-nifU*^*Av*^, then with *Xba*I and *Sac*I to insert *su9-nifS*^*Av*^, and finally digested with *Xho*I to insert *su9-nifM*^*Av*^ by homologous recombination^44^.

All DNA digestions were performed using enzymes from New England Biolabs. Ligated products (T4 ligase, Promega) were introduced into *E. coli DH5α* chemically competent cells and selected on LB (Lysogenic broth) supplemented with appropriate antibiotics. Plasmid extraction was performed using Qiaprep Spin Miniprep kit (QIAGEN) and correct cloning was confirmed by Sanger sequencing (Macrogen).

### Growth of *S. cerevisiae*, mitochondria isolations, and *Sc*NifH purifications

*S. cerevisiae* for galactose-induced expression of *Sc*NifH^*Mm*^*, Sc*NifH^*Mi*^*, Sc*NifH^*Ht*^, and *Sc*NifH^*Av*^ together with SU9-NifU^*Av*^, SU9-NifS^*Av*^, and SU9-NifM^*Av*^ (XJ1Y-XJ4Y, Supplementary Table 3) were cultured in 4-l fermenters under aerobic conditions (0.625 l of air per minute and l of culture, 250 rpm stirring) and used for mitochondria isolations or NifH purifications as previously described^11^. Preparation of CFE and STAC purifications were performed at O_2_-levels below 1 ppm in anaerobic chambers (Coy systems or MBraun). Typically, cells were resuspended in lysis buffer (100 mM Tris-HCl (pH 8.6), 200 mM NaCl, 10% glycerol, 2 mM sodium dithionite (DTH), 1 mM PMSF, 1 μg/ml leupeptin, 5 μg/ml DNAse I) at a ratio of 1:2 (w/v). Total extracts (TE) were prepared by lysis of the cell suspensions under anaerobic atmosphere using an EmulsiFlex-C5 homogenizer (Avestin Inc.) operating at 20,000 psi. The TE was transferrred to centrifuge tubes equipped with sealing closures (Beckman Coulter) and centrifuged at 50,000 *g* for 1 h at 4 °C (Avanti J-26 XP). The supernatant was filtered using filtering cups with a pore size of 0.2 μm, rendering cell-free extract (CFE) of soluble proteins that was loaded at 2.5 ml/min into a 5 ml Strep-Tactin XP column (IBA LifeSciences) attached to an ÄKTA FPLC (GE Heathcare). The column was washed using 75 ml washing buffer (100 mM Tris-HCl pH 8.0, 200 mM NaCl, 10% glycerol, 2 mM DTH). Strep-Tactin XP column-bound proteins were eluted with 15 ml washing buffer supplemented with 50 mM biotin (IBA LifeSciences). The elution fraction was concentrated, and biotin removed, by passing the protein through PD-10 desalting columns (GE Healthcare). Desalted eluate was further concentrated using centrifugal filters (Amicon, Millipore) with 30 kDa cutoff. Finally, the concentrated protein was snap-frozen in cryovials (Nalgene) and stored in liquid N_2_.

### Soil Fe fertilization, preparation of anaerobic *N. benthamiana* leaf cell-free extracts, and purification of *Nb*NifH^*Ht*^ and *Nb*NifU^*Av*^

*N. benthamiana* plants were grown under long day conditions (16 h light/8 h dark) with supporting light from 17:00 to 00:00 for 4 weeks. For Fe fertilization experiments, plants were irrigated (2l per week) with tap water supplemented with 1 g/l Sequestrene G100 (Syngenta). Leaves harvested after extended dark period (16 h) were kept in darkness from 17:00 (previous day) until sample collection (09:00 following morning).

Purifications of *Nb*NifH^*Ht*^ and *Nb*NifU^*Av*^ were performed at O_2_-levels below 1 ppm inside anaerobic chambers (Coy systema or MBraun). Typically, 200 g of leaf material was harvested and frozen in liquid N_2_. Leaf material was transferred into an anaerobic chamber in frozen condition and disrupted in equal amount (w/v) of lysis buffer (100 mM Tris-HCl pH 8.6, 200 mM NaCl, 10% glycerol, 2 mM DTH, 1 mM PMSF, 1 μg/ml leupeptin, 5 μg/ml DNAseI) using a blender (Oster Classic 4655) operating at maximum power and maintained at 4 °C using a circulating water bath. TE was filtered through cheese cloth to remove larger debris. Preparation CFE by centrifugation, Strep-Tactin affinity chromatography, protein elution, concentration, and storage was identical as for yeast-expressed *Sc*NifH proteins. The purification procedure for *Nb*NifU^*Av*^ only differed in that no DTH was present in the buffers.

### Protein methods, antibodies, UV–vis absorption spectrum, and electron paramagnetic resonance

Protein concentrations were measured using the BCA protein assay (PIERCE) in combination with iodoacetamide to eliminate the interfering effect of DTH^45^. Colorimetric Fe determination was performed as reported^46^, and the N-terminal amino acid sequences were determined by Edman degradation (Proteome Factory AG).

Antibodies used in this study and their dilutions for immunoblotting were as follows: polyclonal antibodies detecting NifU^*Av*^ (used at 1:2,000 in 5% BSA), NifS^*Av*^ (used at 1:1,000 in 5% BSA), NifH^*Av*^ (used at 1:5,000 in 5% BSA), NifM^*Av*^ (used at 1:2,000 in 5% BSA) were raised against purified preparations of the corresponding *A. vinelandii* proteins (generated *in house*). Strep-tag II (“Strep-MAB”, 2-1507-001, IBA Lifesciences, 1:2,000 in 5% BSA), Strep-Tactin conjutaged to HRP (“Strep-HRP”, 2-1502-001, IBA Lifesciences, 1:50,000 in TBS- T), GFP (sc-9996, Santa Cruz Biotechnology, 1:2,000 in 5% BSA), HSP60 (LK-2, ab59458, Abcam, 1:1,000 in 5% BSA), and Tubulin (3H3087, sc-69971, Santa Cruz Biotechnology, 1:500 in 5% BSA) specific antibodies are commercially available.

The UV–vis absorption spectra were recorded after removal of the DTH from the protein samples using PD-10 desalting columns (GE Healthcare) equilibrated with the corresponding protein buffer wihtout DTH. DTH-free protein samples were then diluted in the same buffers and transferred to a Q6 spectroscopy cuvettes with sealing closures. Absorption (280 nm to 800 nm) was recorded using a UV-2600 spectrophotometer (Shimadzu).

EPR measurements were performed in a Bruker E500 spectrometer equipped with a resonator operating in the TE_102_ mode at 9.47 GHz. Temperature was set and stabilized to 10 K by an Oxford temperature controller regulating a gas-flow cryostat refrigerated with helium. For measurements, a microwave power of 2.5 mW and a magnetic field modulation amplitude of 1 mT was used. Experimental conditions were carefully monitored to avoid over-modulation or saturation effects. Simulations of the EPR spectra were performed using the Matlab toolbox Easyspin^47^.

### In vitro NifH activity

NifH activity was determined as described by Shah et al. with slight modifications^48^. Reactions were prepared inside anaerobic chambers. Purified NifH proteins were analyzed by ARA after addition of NifDK^*Av*^ and ATP-regenerating mixture (1.23 mM ATP, 18 mM phosphocreatine, 2.2 mM MgCl_2_, 3 mM DTH and 46 μg/ml of creatine phosphokinase, 22 mM Tris-HCl pH 7.5) in a final volume of 600 μl inside 9 ml serum vials under Ar atmosphere containing 500 μl of acetylene (1 atm). The ratio of NifH to NifDK in the assays was 40:1 unless otherwise indicated. The ARA were performed at 30 °C in a shaking water bath for 15 min. Reactions were stopped by adding 100 μl of 8 M NaOH. Positive control reactions for acetylene reduction were carried out with NifH^*Av*^. Ethylene formed was measured in 50 μl gas phase samples using a Porapak N 80/100 column in a gas chromatograph (Shimadzu).

Reduction of N_2_ to NH_3_ was determined in reaction mixtures prepared as for the ARA but containing 100 mM 3-(N-morpholino)propanesulfonic acid (MOPS), pH 7.8, as buffer. Mixtures were prepared in volumes of 750 μl, from which 100 μl was removed at assay start to serve as background (*t*_o_) for NH_3_ measurements. After exchanging vial atmosphere for N_2_, mixtures were incubated at 30 °C for 30 min, and reactions were stopped by addition of 100 μl 5 M EDTA. Twenty-five μl of the blank (*t*_o_) and the reaction (*t*_30_) were added in duplicates to 200 μl o-phthaldialdehyde reagent solution (ThermoFisher Scientific) in 96-well microplate for fluorescence-based assays (Nunc). Fluorescence (Ex 390 nm, Em 472 nm) was measured using a Varioskan LUX plate reader (ThermoFisher Scientific). NH_3_ production was determined from the increase in fluorescence (*t*_30_-*t*_o_) against standards prepared with NH_4_Cl and recorded in the same plate.

### In vitro P-cluster maturation

P-cluster maturation assays were performed inside anaerobic chambers. The in vitro assay combined isolated NifH to be tested (50 μg) with *A.vinelandii* DJ77 (Δ*nifH*) CFE (4.34 mg total protein) and an excess of pure FeMo-co (0.85 μM) in 500 μl ATP-regenerating mixture as described above. Reactions were incubated at 30 °C for 30 min. Forty μl of 1 mM (NH_4_)_2_MoS_4_ (tetrathiomolybdate) were then added and mixtures were incubated for 10 min at room temperature to prevent further FeMo-co incorporation into NifDK^*Av*^ during the ARA.

Apo-NifDK^*Av*^ activation after P-cluster maturation and FeMo-co insertion was analyzed by ARA after addition of an excess of the same NifH species (100 μg) and ATP-regenerating mixture in a final reaction volume of 1 ml. ARA was carried out in 9 ml serum vials containing Ar and 500 μl of acetylene (1 atm) in the headspace for 15 min at 30 °C. Positive control reactions for in vitro P-cluster maturation and ARA contained purified NifH^*Av*^. Ethylene formed was measured in 50 μl gas phase samples using a Porapak N 80/100 column in a gas chromatograph (Shimadzu).

### In vitro FeMo-co synthesis and apo-NifDK^*Av*^ reconstitution

NifB-co-dependent FeMo-co synthesis assays were performed inside anaerobic chambers as described by Curatti et al., with slight modifications^34^. One hundred μl reactions contained 3.0 μM NifH, GST-NifX-NifB-co (20.4 μM Fe), 1.5 μM apo-NifEN^*Av*^, 0.6 μM apo-NifDK^*Av*^, 17.5 μM Na_2_MoO_4_, 175 μM *R*-homocitrate, 1 mg/ml BSA, and ATP-regenerating mixture (1.23 mM ATP, 18 mM phosphocreatine disodium salt, 2.2 mM MgCl_2_, 3 mM DTH, 46 μg/ml creatine phosphokinase, final concentrations in 22 mM Tris-HCl (pH 7.5) buffer at 30 °C for 60 min.

NifB-dependent FeMo-co synthesis assays were performed as the above described NifB-co-dependent assay replacing GST-NifX-NifB-co by 10.0 μM NifB monomer, 125 μM FeSO_4_, 125 μM Na_2_S, and 125 μM SAM.

Following in vitro synthesis of FeMo-co, 17.5 μM (NH_4_)_2_MoS_4_ was added to prevent further FeMo-co incorporation into apo-NifDK^*Av*^, and incubated for 10 min at 25 °C. Activation of apo-NifDK^*Av*^ was analyzed by addition of 500 μl ATP-regenerating mixture and *Sc*NifH^*Ht*^ (2.0 μM final concentration) in 9 ml vials containing Ar and 500 μl acetylene. The ARA were performed at 30 °C for 20 min. Positive control reactions for ARA contained NifDK^*Av*^ and NifH^*Av*^. Ethylene formed was measured in 50 μl gas phase samples using a Porapak N 80/100 column in a gas chromatograph (Shimadzu).

### In vitro [Fe-S] cluster reconstitution and NifH activity

In vitro [Fe-S] cluster reconstitutions of NifH and NifU^*Av*^ purified from *E. coli* (*Ec*NifU^*Av*^)^10^ were performed in anaerobic chambers as described by Zheng and Dean^49^ with slight modifications. NifH or NifU (20 μM) was added to 22 mM Tris-HCl (pH 7.5) buffer supplemented with 8 mM 1,4-dithiothreitol (DTT) in a final volume of 100 μl and incubated at 37 °C for 30 min. Then, reactions were supplemented with 1 mM L-cysteine, 1 mM DTT, 400 μM (NH_4_)_2_Fe(SO_4_)_2_, and 225 nM NifS^*Av*^ purified from *E. coli* (*Ec*NifS^*Av*^)^10^, and incubated at 37 °C overnight. Finally, the proteins were diluted 1000-fold in 22 mM Tris-HCl (pH 7.5) buffer, and then concentrated using centrifugal filters (Amicon, Millipore) with 30 kDa cutoff to remove excess reagents.

For “direct reconstitution” activity assays, the activity of [Fe_4_S_4_] cluster reconstituted NifH protein was determined using ARA. For “NifU-mediated reconstitution”, as-isolated NifH protein was mixed with [Fe-S] cluster reconstituted *Ec*NifU^*Av*^, and then immediately used for ARA.

### Statistics and reproducibility

Distinct samples were used for in vitro activity measurements and sample sizes are indicated by *n*, where each distinct sample was measured at least two times. Mean of measured activities are shown. The data presented in the figure graphs are listed in Supplementary Data 2.

## Supplementary information

Supplementary Information

Description of Additional Supplementary Files

Supplementary Data 1

Supplementary Data 2

## Data Availability

The authors declare that the data supporting the findings of this study are available within the article, its supplementary information and data, and upon request.
