# Supplementary material for: Exploiting genetic diversity and gene synthesis to identify superior nitrogenase NifH protein variants to engineer N2-fixation in plants
Source: Commun Biol. 2021 Jan 4;4:4. doi: 10.1038/s42003-020-01536-6 (PMC7782807; doi:10.1038/s42003-020-01536-6)
Supplement: Supplementary file 1 — Supplementary Information [file 42003_2020_1536_MOESM1_ESM.pdf]

SUPPLEMENTARY INFORMATION

Title

Exploiting genetic diversity and gene synthesis to identify superior nitrogenase NifH protein variants to engineer N_2-fixation in plants

Authors

Xi Jiang[1,2], Lucía Payá-Tormo[1,2], Diana Coroian[1], Inés García-Rubio[3], Rocío Castellanos-Rueda[1,d], Álvaro Eseverri[1,2], Gema López-Torrejón[1,2], Stefan Burén[1], and Luis M. Rubio[1,2]

Affiliations

[1] Centro de Biotecnología y Genómica de Plantas, Universidad Politécnica de Madrid, Instituto Nacional de Investigación y Tecnología Agraria y Alimentaria, Pozuelo de Alarcón, 28223 Madrid, Spain.

[2] Departamento de Biotecnología-Biología Vegetal, Escuela Técnica Superior de Ingeniería Agronómica, Alimentaría y de Biosistemas, Universidad Politécnica de Madrid, 28040 Madrid, Spain.

[3] Centro Universitario de la Defensa, Ctra. de Huesca s/n, 50090 Zaragoza, Spain.

Present addresses:

Rocío Castellanos-Rueda: Department of Biosystems Science and Engineering, ETH Zürich, 4058 Basel, Switzerland.

Author ORCID numbers

Xi Jiang (0000-0002-1819-9041), Lucía Payá-Tormo (0000-0003-0862-5235), Inés García Rubio (0000-0002-1827-1250), Rocío Castellanos-Rueda (0000-0002-9674-4172), Álvaro Eseverri (0000-0002-7005-6742), Stefan Burén (0000-0002-8487-2732), and Luis M. Rubio (0000-0003-1596-2475).

[1] Corresponding Authors

Stefan Burén (0000-0002-8487-2732), Luis M. Rubio (0000-0003-1596-2475)
Centro de Biotecnología y Genómica de Plantas, Universidad Politécnica de Madrid, Instituto Nacional de Investigación y Tecnología Agraria y Alimentaria, Pozuelo de Alarcón, 28223 Madrid, Spain.
stefan.buren@upm.es, lm.rubio@upm.es

Supplementary Methods

Preparation of *A. tumefaciens* for infiltration of *nifH* library in tobacco leaves

Heat-shock competent *A. tumefaciens* GV3101 were prepared using standard protocols. All modified plasmids carrying mitochondria-targeted *cox4-ts-nifH* and *gfp* (pN2XJ81-pN2XJ112), mitochondria-targeted *su9-nifU*, *su9-nifS*, and *su9-nifM* (pN2XJ165), or the *p19* silencing suppressor GB1203[1] (GoldenBraid 2.0, plasmid #68214, Addgene) were individually introduced by transformation into *A. tumefaciens* GV3101 before each leaf-infiltration experiment. Plasmid maintenance was selected on solid LB medium supplemented with 10 μg/ml gentamicin (Sigma-Aldrich), 25 μg/ml rifampicin (Sigma-Aldrich), and 50 μg/ml kanamycin (FORMEDIUM).

Two days before tobacco leaf-infiltration, single *A. tumefaciens* colonies were used to start precultures of 5 ml in LB with the appropriate antibiotics. Precultures were incubated in a shaker at 150 rpm and 28°C overnight. One hundred μl of these precultures were used to inoculate cultures of 50 ml, which were then incubated at 150 rpm and 28°C overnight. The day of infiltration, each *A. tumefaciens* GV3101 culture (normally at an OD_{600nm} of 3-4) was induced for 2 h at room temperature without shaking in 10 mM Mg_2SO_4, 10 mM 2-(N-Morpholino)ethanesulfonic acid (MES), and 150 μM acetosyringone at a final OD_{600nm} of 0.9. Then, each induced *A. tumefaciens* GV3101 culture containing pN2XJ81-pN2XJ112 was mixed in equal parts (1:1:1) with cultures containing pN2XJ165 and cultures containing GB1203, rendering mixtures at OD_{600nm} 0.9. Syringes of 1 ml were used to infiltrate mixtures into 4-week old tobacco leaves. In total, 32 mixtures were prepared for expression of the 32 *nifH* variants and GFP, together with SU9-NifU, SU9-NifS, SU9-NifM, and p19. Each mixture was prepared and infiltrated in three independent experiments.

Preparation of total and soluble tobacco protein extracts for *Nb*NifH^{Xx} library screening

Four days after infiltration, 10 leaf disks (5 mm diameter) were excised from leaves, transferred to 2 ml Eppendorf tubes containing a steel ball (7 mm diameter), and snap-frozen in liquid N_2. Leaf tissue was ground using a lab vibration mill mixer (QIAGEN Retsch MM300 TissueLyser) for 1 min at 30 Hz. For total extracts, 400 μl of 2x Laemli buffer was added to ground leaf tissue and heated at 95°C for 10 min. For soluble protein extracts, 400 μl of protein extraction buffer[2] was immediately added to each tube before incubation by inversion using a spinning wheel for 30 min at 4°C. Cell debris was removed by centrifugation at 14000 rpm for 30 min at 4°C in a benchtop centrifuge. Finally, the supernatant was transferred to a new tube and prepared for immunoblot analysis by mixing with equal volume of 2x Laemmli buffer.

Cloning in yeast-expression vectors

The pESC-His (Agilent) vector was used to generate pN2XJ187 – pN2XJ190. First the *su9-nifM* was amplified from pN2XJ165 using primers that introduced *BamH*I (AGGAGAAAAAACCCCGGATCCCTATCTCTCTCGAGATGGC) and *Xho*I (GCGGTACCAAGCTTACTCGAGGGAGAAACTCGAGTTAACC) sites and was inserted into pESC-His generating pN2XJ186. The *cox4-ts-nifH*^{Xx} constructs were then amplified from pN2XJ83 (*cox4-ts-nifH*^{Av}), pN2XJ105 (*cox4-ts-nifH*^{Mm}), pN2XJ106 (*cox4-ts-nifH*^{Mi}), and pN2XJ108 (*cox4-ts-nifH*^{Ht}) using primers introducing *EcoR*I (AATTTTTGAAAATTCGAATTCCTCTTGACCATGCTTTCAC) and *Sac*I

(GAAGAATTGTTAATTAAGAGCTCGGGGAAATTCGAGCTGG) sites, and inserted into pN2XJ186, generating pN2XJ190, pN2XJ188, pN2XJ189, and pN2XJ187, respectively.

***In vivo* NifH complementation in *A. vinelandii* DJ77**

Procedures for *A. vinelandii* growth and transformation have been described[3]. The *ts-nifH*^{Ht} gene was codon-optimized for expression in *K. oxytoca* and cloned (Genscript) in pUC58 using *Nco*I and *Not*I sites. The *ts-nifH*^{Ht} gene was amplified by PCR with primers (AATGCAACCTGAGGAAATTACATATGGCGTGGAGCCACCC, GCTTGATATCGAATTCGGTCACCCCGCTCACGCCACTTCC) introducing *Nde*I and *EcoR*I sites and inserted into pRHB272[4] digested with *Nde*I and *EcoR*I to generate pN2XJ202, in which *ts-nifH*^{Ht} is under *nifH* promoter control and prepared for recombination in the chromosomal region downstream of Avin02530. *A. vinelandii* DJ77 (Δ*nifH*) was transformed with pN2XJ202 to generate strain UW481. Transformants were selected by resistance to ampicillin followed by growth on N-free Burk medium. Colonies presenting Nif$^+$ phenotype were analyzed by colony PCR to ensure presence of the *ts-nifH*^{Ht} insert. Similar procedures were previously used to generate strain UW480 (DJ77 derivative carrying P_{nifH}::*ts-nifH*^{Av})[5], in which the *ts* sequence (codon-optimized for expression in *S. cerevisiae*) was added to the *A. vinelandii nifH* sequence.

For diazotrophic growth determinations, strains were cultured (in duplicates) in 50 ml Burk complete medium at 200 rpm, 30°C, overnight. Cultures were then used to inoculate 50-ml Burk complete media at OD_{600nm} of 0.4 and were incubated at 200 rpm, 30°C, until reaching OD_{600nm} of 1. These cultures were then used to inoculate at an OD_{600nm} of 0.6: i) 30 ml Burk complete medium and ii) 30 ml Burk medium without N source, which were then incubated at 200 rpm and 30°C. Growth (OD_{600nm}) and *in vivo* nitrogenase activity by ARA were determined every 2-4 h. For ARA measurements, 1 ml of each culture was transferred to a 9 ml serum vial containing air in the headspace. After injection of 500 μl acetylene, vials were incubated in a water bath for 15 min at 30°C. Ethylene formed was measured in 50 μl gas phase samples using a Porapak N 80/100 column in a gas chromatograph (Shimadzu).

For preparation of *A. vinelandii* total protein extracts, 1 ml culture was centrifuged at 13,000 rpm in a benchtop centrifuge for 2 min and the supernatant was carefully discarded. The pellet was resuspended in 50 μl of 2x Laemmli buffer (OD^{-1} ml^{-1}) and heated at 95°C for 10 min.

Generation of pN2XJ198 for purification of *Nb*NifU^{Av}

*Nb*NifU^{Av} purification vector was constructed by modification of plasmid pN2SB41. First, *p19* gene was amplified using primers containing *Xho*I (ACAAATCTATCTCTCTCGAGATGGAACGAGCTATACAAGG, ATTATTATGGAGAAACTCGAGTCACTCGCTTTCTTTTTCG) sites and homologous sequences to the flanking region of *Xho*I in pN2SB41. The used template was GB1203. This PCR product was used to replace the *hygR* by digesting with *Xho*I and inserted into the backbone by homologous recombination[6]. Then, *nifU* gene was amplified using primers with *BamH*I (CCACAATTTGAAAAAGGATCCTGGGACTACTCTGAAAAGG) and *BstE*II (AAAAAAGGTCACCTTAGACTTCCATTTGGGCGTGTGCG) sites to insert it in the modified pN2SB41 (Δ*hygR* p35S::*p19*) vector by replacing the *gus* gene flanked by the same restriction sites to generate pN2XJ178. Finally, *su9-nifS* gene was inserted by digesting pN2XJ178 and pN2XJ165 with *EcoR*I and *BspE*I, purifying the 11-kb and 2-kb fragment, respectively, and ligating them to generate pN2XJ198 (Supplementary Table 1).

Generation of pAE382 for purification of *Nb*NifH^{Ht}

The multigene construct containing *H. thermophilus nifH* and *A. vinelandii nifM, nifU* and *nifS* together with the silencing suppressor p19[7] and eGFP was generated using MoClo[8, 9]. Primers for Level 0 modular pieces were designed using Domesticator Tool[1] (https://gbcloning.upv.es/do/domestication/) and subsequently modified to add a *Bpi*I site. Fragments containing internal *Bsa*I or *Bpi*I sites were mutated to remove those restriction sites using internal primers. Modular pieces were amplified by PCR using Phusion Hot Start II DNA Polymerase (ThermoFisher).

MoClo reactions were performed at 2:1 molar ratio of insert to acceptor vector in 20 μl reactions containing 5 U of the required restriction enzyme (*Bpi*I (Thermo Scientific) for modular pieces and multigene constructs, and *Bsa*I-HFv2 (New England Biolabs) to generate transcriptional units), 4.5 U of T4 Ligase, 1.5 μl of 10x ligation buffer (Promega) and 1.5 μl of 10x BSA (Canvax Biotech). Reactions were incubated in a thermocycler using the following program: 20 sec at 37°C, 26 cycles of (3 min at 37°C and 4 min at 16°C), 5 min at 50°C, 5 min at 80°C and finally held at 16°C.

Chemically competent *E. coli* DH5α cells were transformed with DNA from reaction mixtures and then grown in solid LB medium supplemented with 20 μg/ml X-Gal (Duchefa Biochemie), 1 mM IPTG (Sigma-Aldrich), and 50 μg/ml spectinomycin (Sigma-Aldrich) for Level 0 constructs, 100 μg/ml carbenicillin (Formedium) for Level 1 constructs, or 100 μg/ml kanamycin (Formedium) for Level 2 constructs. White colonies were selected for plasmid DNA extraction using GenElute Plasmid Miniprep Kit (Sigma-Aldrich). Fidelity of all DNA constructs was verified by Sanger sequencing (Eurofins genomics).

3D-modelling of NifH variants

3D-models of NifH variants were generated by homology modelling (http://swissmodel.expasy.org/)[10] using *A. vinelandii* NifH structure (PDB: 1G1M).

Supplementary Figures

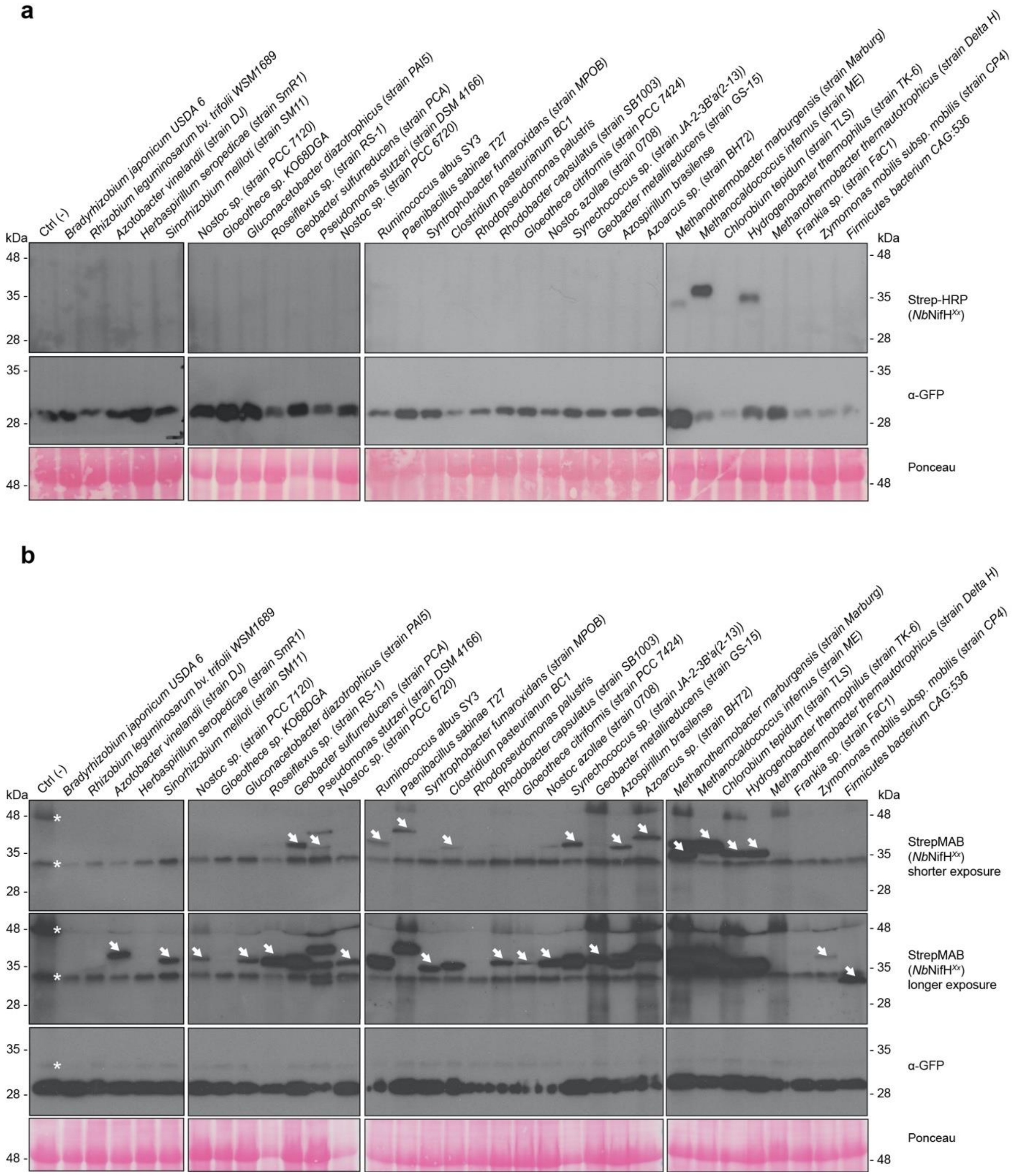

Supplementary Figure 1 Screening of *Nb*NifH^{Xx} solubility in tobacco mitochondria. (**a**-**b**) Immunoblots showing accumulation of *Nb*NifH^{Xx} and GFP (infiltration control) in soluble (a) or total (b) protein extracts of *A. tumefaciens* infiltrated tobacco leaves. White arrows indicate the expected full-size *Nb*NifH^{Xx} proteins upon processing of the COX4 mitochondria signal, according to the sizes indicated in Supplementary Table 1. White stars indicate non-specific signals also seen in the GFP control lane. All *N. benthamiana* plants were co-infiltrated with a mixture of *A. tumefaciens* strains containing plasmids for expression of *Nb*NifU^{Av}, *Nb*NifS^{Av}, and *Nb*NifM^{Av} (pN2XJ165) and p19 (GB1203), in addition to the *Nb*NifH^{Xx} variants and GFP

(plasmids pN2XJ81-pN2XJ112). The figure shows representative immunoblots of three independent infiltration experiments. Uncropped immunoblots and membranes are shown in Supplementary Fig. 11 and 12.

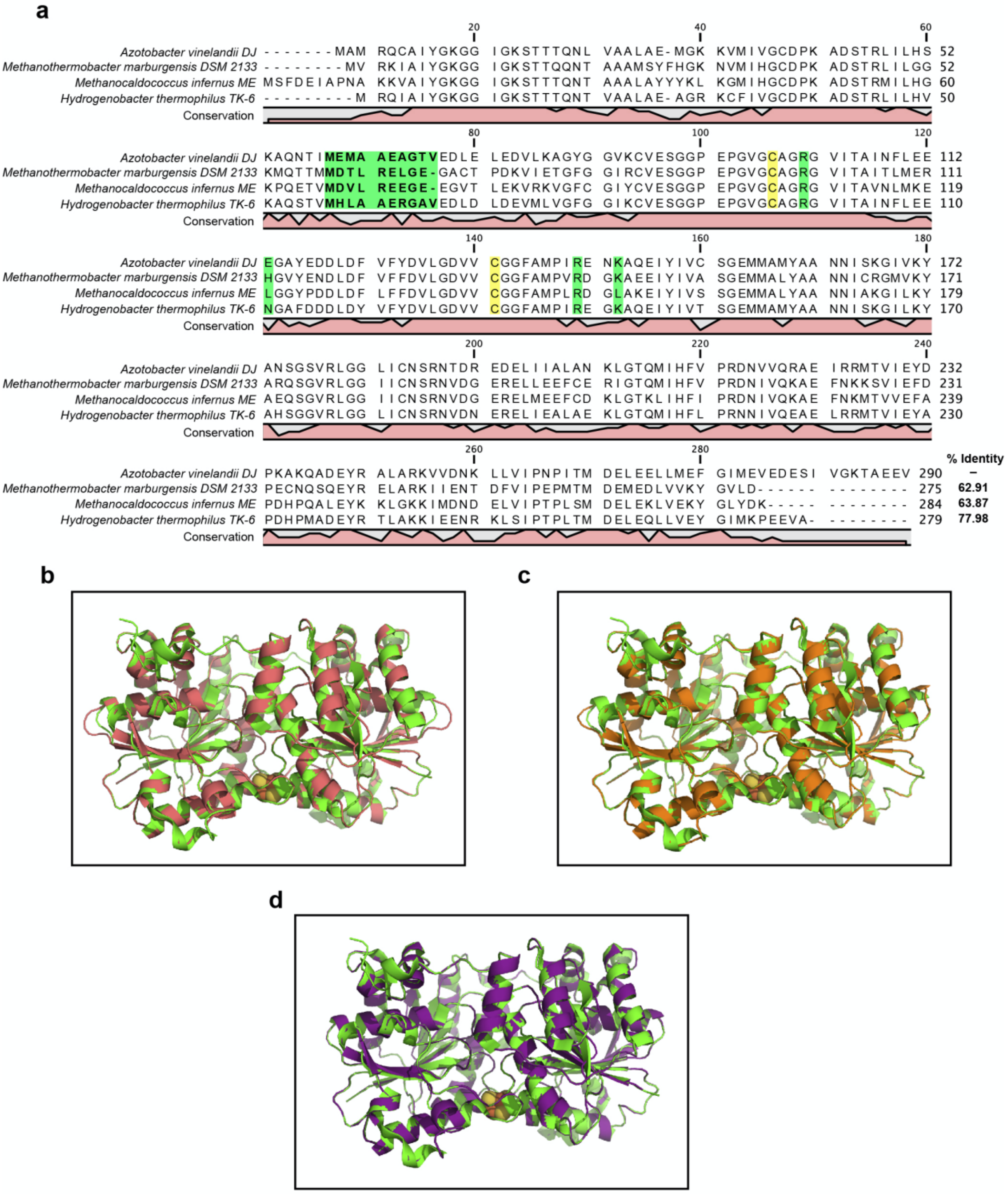

Supplementary Figure 2 Comparison of selected candidates with NifH^{Av}. (**a**) Sequence alignments using Clustal Omega[11]. Conserved Cys coordinating the [4Fe-4S] clusters are highlighted in yellow. NifH^{Av} residues positions (green) and loop region (bold) involved in interaction with NifDK^{Av} are highlighted[12]. The percentage identity is calculated using blastp[13] using the NifH^{Av} sequence as query. (**b-d**) Overlay of 3D-structural models for *M. marburgensis* NifH (b, pink), *M. infernus* (c, orange), or *H. thermophilus* (d, purple) with *A. vinelandii* (green). All three proteins are expected to form homodimers with a [4Fe-4S] cluster at the subunit interface.

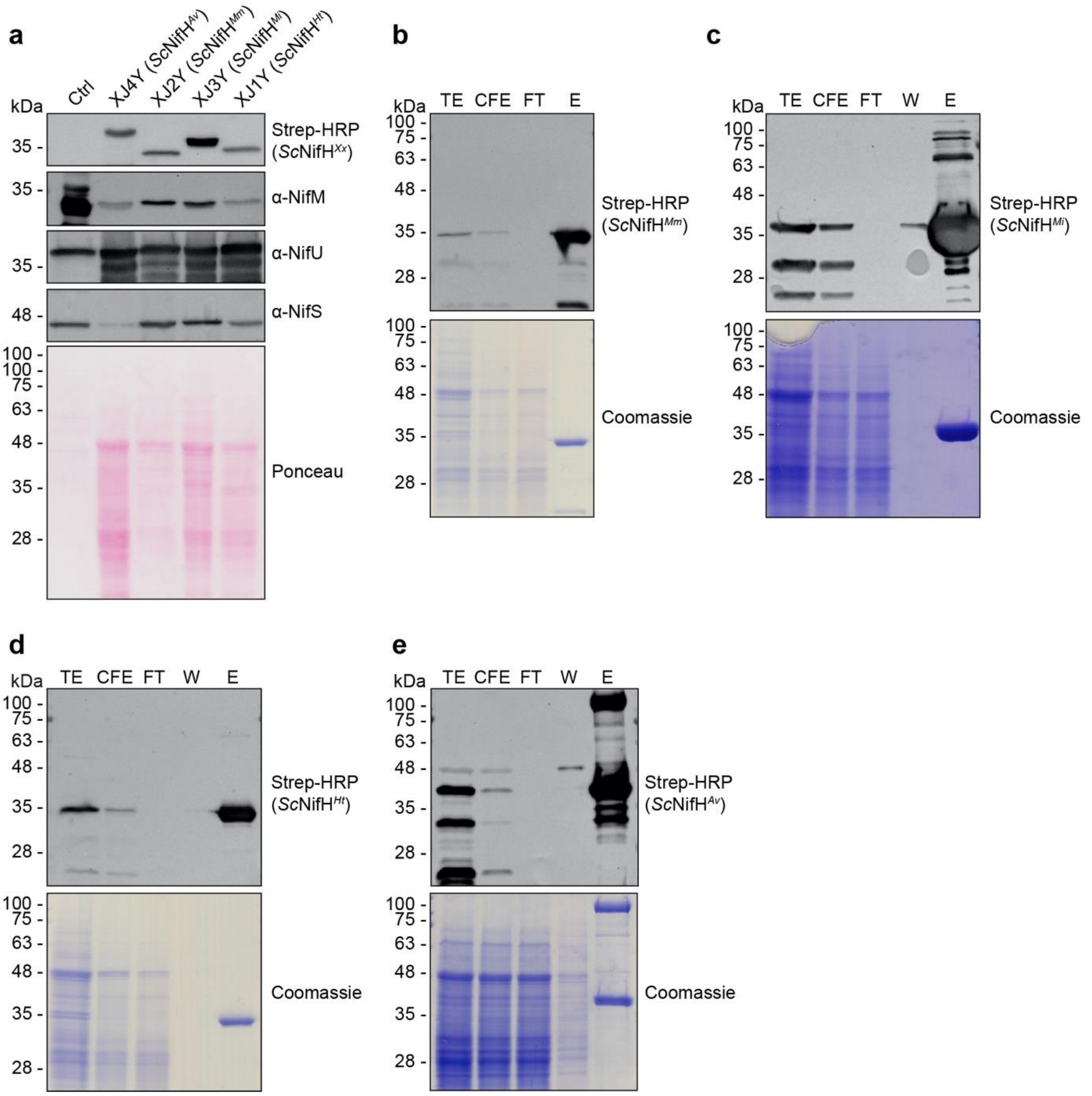

Supplementary Figure 3 Purification of selected *Sc*NifH^{Xx} variants from mitochondria of aerobically cultured *S. cerevisiae*. (**a**) Immunoblots showing the accumulation of *Sc*NifH^{Xx} proteins, and *Sc*NifU^{Av}, *Sc*NifS^{Av}, and *Sc*NifM^{Av} in total extracts from yeast strains XJ4Y (expressing *Sc*NifH^{Av}), XJ2Y (expressing *Sc*NifH^{Mm}), XJ3Y (expressing *Sc*NifH^{Mi}), and XJ1Y (expressing *Sc*NifH^{Ht}). CFE from *A. vinelandii* DJ (for NifU and NifS) or *E. coli* strain expressing the *A. vinelandii* NifM were used as size controls. Ponceau staining is shown as loading and transfer control. (**b**-**e**) GAL-induced expression and STAC purification of *Sc*NifH^{Mm} (b), *Sc*NifH^{Mi} (c), ScNifH^{Ht} (d), and *Sc*NifH^{Av} (e). Fractions were analyzed by SDS-PAGE followed by immunoblot and Coomassie staining. TE = total extract, CFE = soluble cell free extract, FT = flow through fraction, W = wash fraction, E = elution fraction. Uncropped immunoblots and gels are shown in Supplementary Fig. 13.

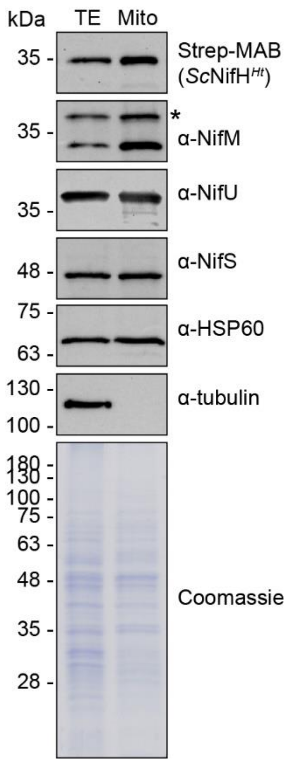

Supplementary Figure 4 Mitochondria targeting of Nif proteins expressed in yeast strain XJ1Y. Immunoblot analysis of total extracts (TE) and mitochondria isolations (Mito) showing mitochondria targeting of *Sc*NifH^{Ht}, *Sc*NifM^{Av}, *Sc*NifU^{Av}, and *Sc*NifS^{Av}. The upper band detected using NifM antibodies (*) could represent SU9-NifM imported into mitochondria but not processed. Antibodies recognizing cytoplasmic (tubulin) and mitochondria (HSP60) control proteins are included. Uncropped immunoblots and gel are shown in Supplementary Fig. 14.

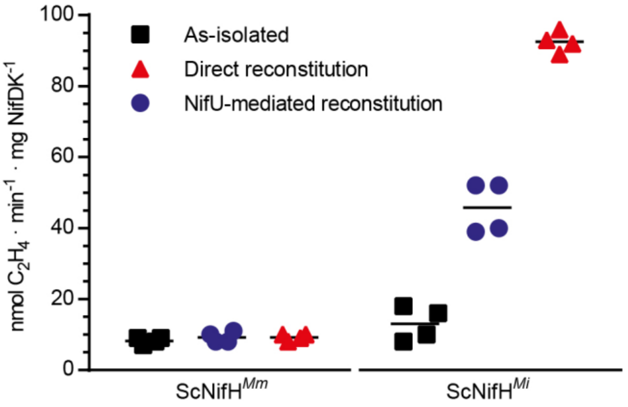

Supplementary Figure 5 Activation of as-isolated *Sc*NifH^{Mm} and *Sc*NifH^{Mi} proteins with [Fe_4S_4] clusters either using Fe, L-cysteine, DTT, and *Ec*NifS^{Av} (direct reconstitution), or [Fe_4S_4] cluster-loaded *Ec*NifU^{Av} (NifU-mediated reconstitution). Control reactions using holo-NifH^{Av} generated 1944 ± 36 (direct reconstitution) and 2116 ± 11 (NifU-mediated reconstitution) units (nmol ethylene formed per min and mg of NifDK^{Av}). Control reactions using apo-NifH^{Av} generated 1948 ± 225 (direct reconstitution) and 2081 ± 197 (NifU-mediated reconstitution) units. Negative control reactions using non-reconstituted apo-NifH resulted in 9 ± 0 units. Data represent mean values (*n* = 4 technical replicates).

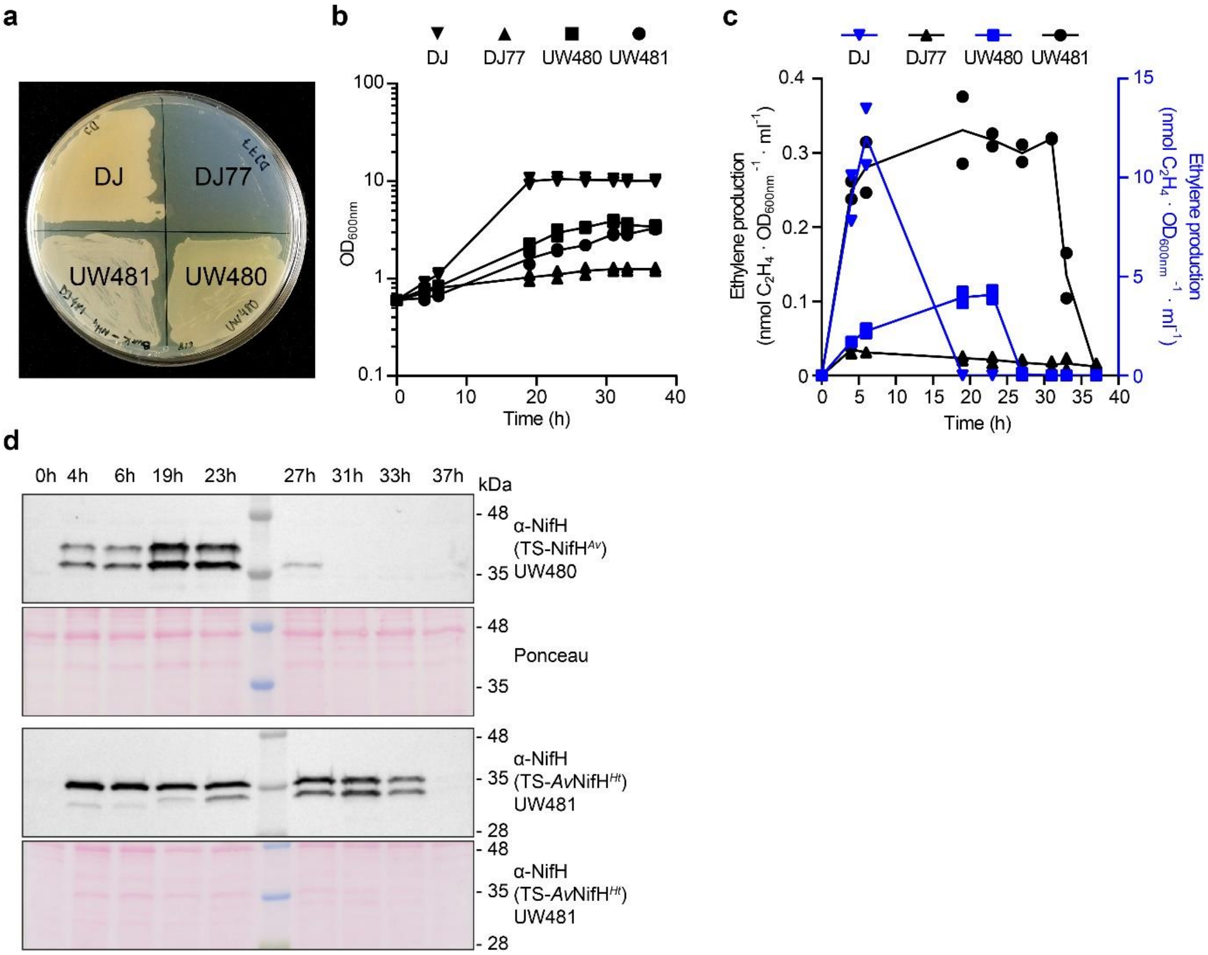

Supplementary Figure 6 *In vivo* functionality of NifH^{Ht}. (**a**) Petri dish containing modified Burk medium without N and showing growth of *A. vinelandii* strains DJ (wild type), DJ77 (Δ*nifH*), UW480 (DJ77 transformed with P_{nifH}::*ts-nifH*^{Av}) and UW481 (DJ77 transformed with P_{nifH}::*ts-nifH*^{Ht}). (**b-c**) Diazotrophic growth (b) and nitrogenase activity (ARA) (c) of *A. vinelandii* strains DJ (wild-type), DJ77 (Δ*nifH*), UW480 (DJ77 transformed with P_{nifH}::*ts-nifH*^{Av}), and UW481 (DJ77 transformed with P_{nifH}::*ts-nifH*^{Ht}) in modified Burk media without N. Data show mean and individual data points (*n* = 2 biological replicates). (**d**) Immunoblot analysis of total protein extracts of *A. vinelandii* UW480 and UW481 from different time-points where time 0 h represents start of derepression. The faster migrating band seen for each protein could result from inefficient translation of the TS-tag, albeit similar NifH SDS-gel doublets are frequent and have been reported [14, 15]. Uncropped immunoblots and membranes are shown in Supplementary Fig. 15.

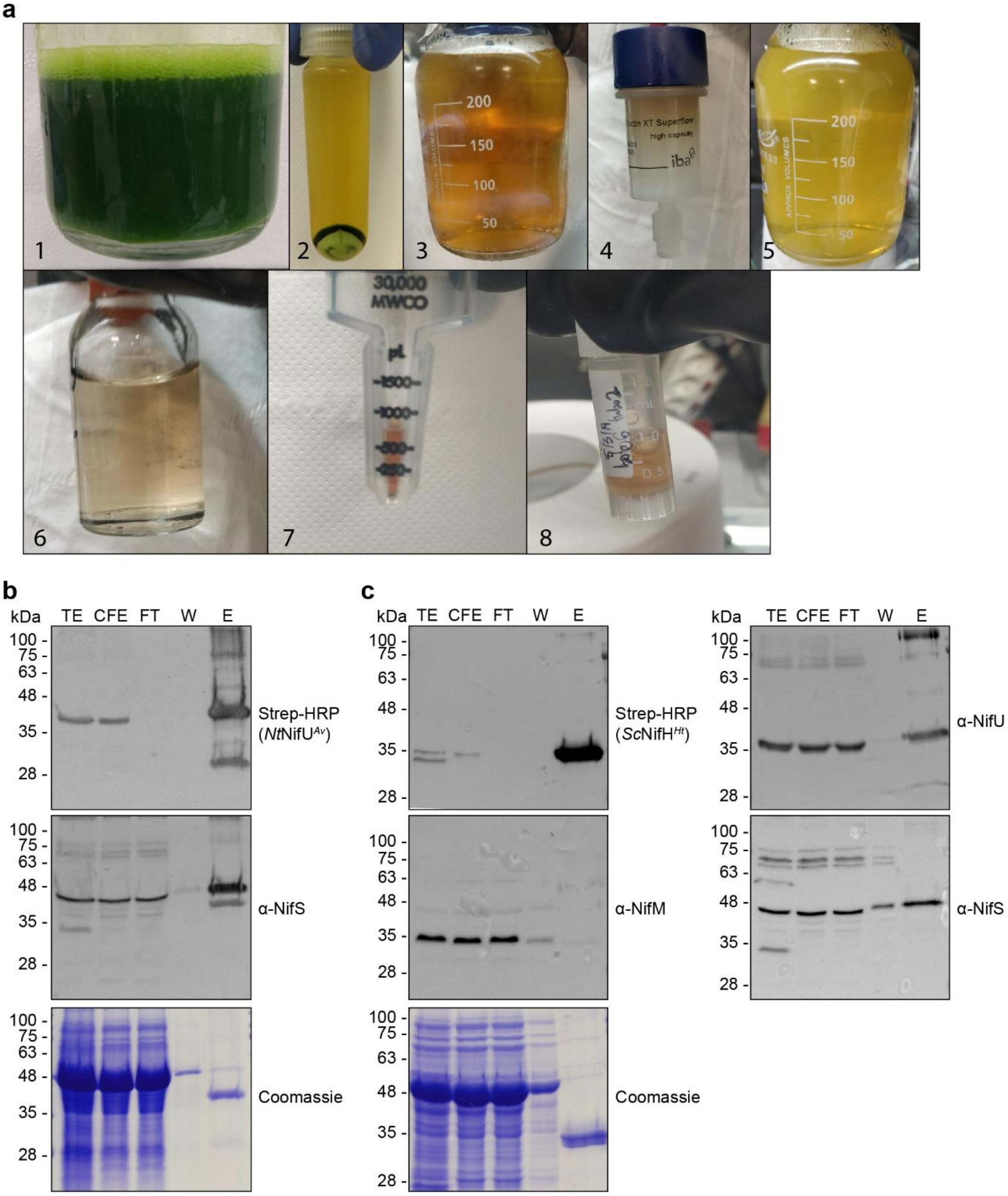

Supplementary Figure 7 STAC purification procedure of mitochondria targeted *Nb*NifU^{Av} and *Nb*NifH^{Ht}. (**a**) Pictures of *Nb*NifU^{Av} purification procedure showing: 1) total tobacco leaf extract, 2) total tobacco leaf extract after centrifugation to separate soluble (supernatant) and insoluble (pellet) fractions, 3) resulting extract after supernatant filtration (denoted CFE), 4) Streptactin XT column loaded with *Nb*NifU^{Av} after passing of CFE, 5) flow-through fraction, 6) biotin-eluted *Nb*NifU^{Av} (about 10 ml), 7) concentrated *Nb*NifU^{Av}, and 8) final *Nb*NifU^{Av} preparation stored in a cryogenic vial. (**b**) STAC purification of *Nb*NifU^{Av} from tobacco leaves infiltrated with *A. tumefaciens* containing plasmid pN2XJ198 (for expression of *Nb*NifU^{Av}, *Nb*NifS^{Av} and p19). (**c**) STAC purification of *Nb*NifH^{Ht} from tobacco leaves infiltrated with *A. tumefaciens* containing plasmid pAE382 (for expression of *Nb*NifH^{Ht}, *Nb*NifM^{Av}, *Nb*NifU^{Av},

*Nb*NifS^{Av}, GFP and p19). Fractions in (b-c) were analyzed by SDS-PAGE followed by immunoblots and Coomassie staining. TE = total extract, CFE = soluble cell free extract, FT = flow through fraction, W = wash fraction, E = elution fraction. Molecular mass markers and antibodies used are indicated to the left and right of each panel, respectively. Uncropped immunoblots and gels are shown in Supplementary Fig. 16.

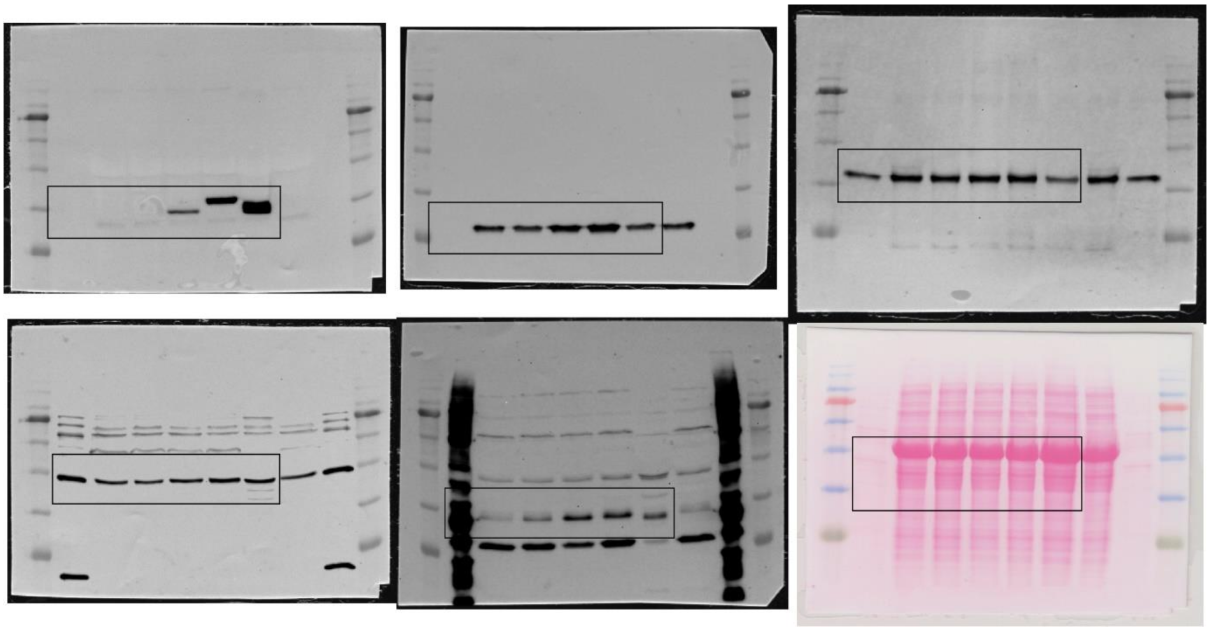

Supplementary Figure 8 Uncropped immunoblots and membrane shown in Figure 1c.

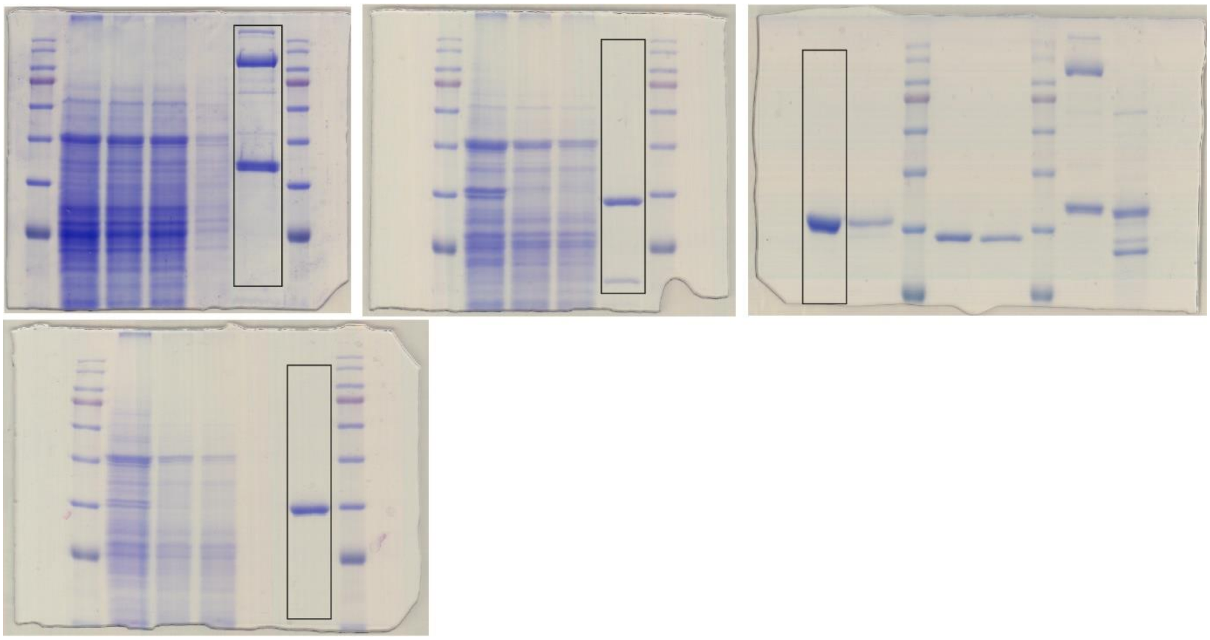

Supplementary Figure 9 Uncropped gels shown in Figure 2a.

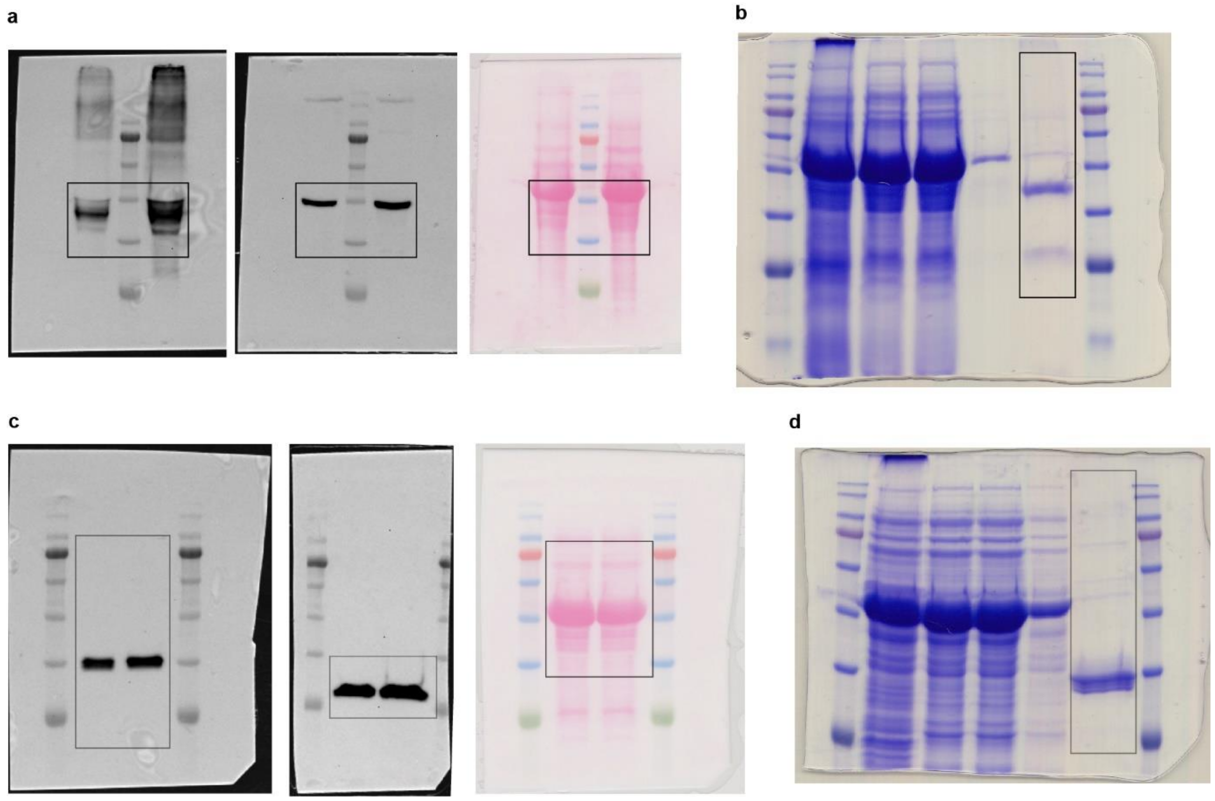

Supplementary Figure 10 Uncropped immunoblots, membranes and gels shown in Figure 4. (**a**) Uncropped immunoblots and membrane shown in Figure 4b. (**b**) Uncropped gel shown in Figure 4c. (**c**) Uncropped immunoblots and membrane shown in Figure 4e. (**d**) Uncropped gel shown in Figure 4f.

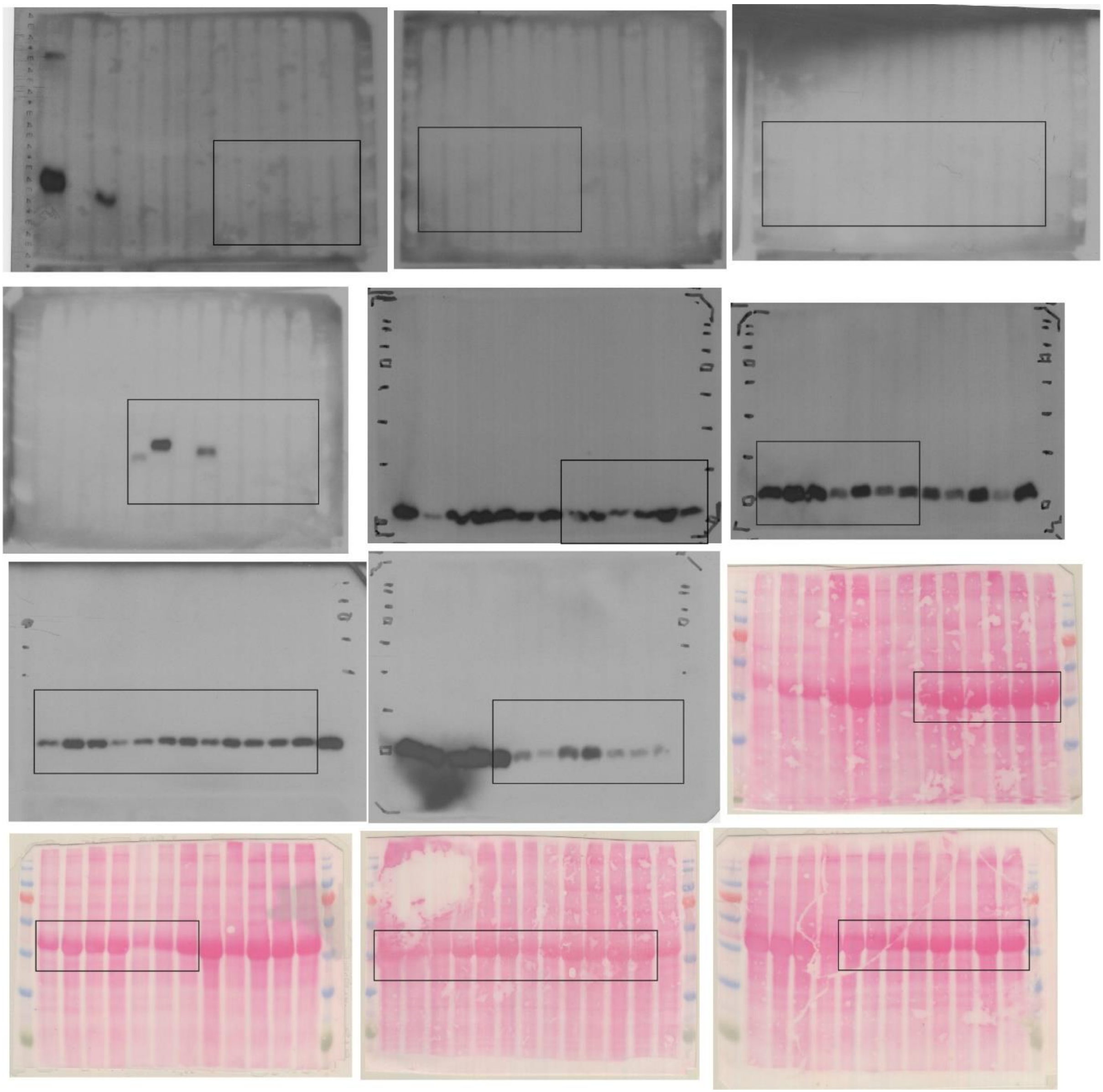

Supplementary Figure 11 Uncropped immunoblots and membranes shown in Supplementary Figure 1a.

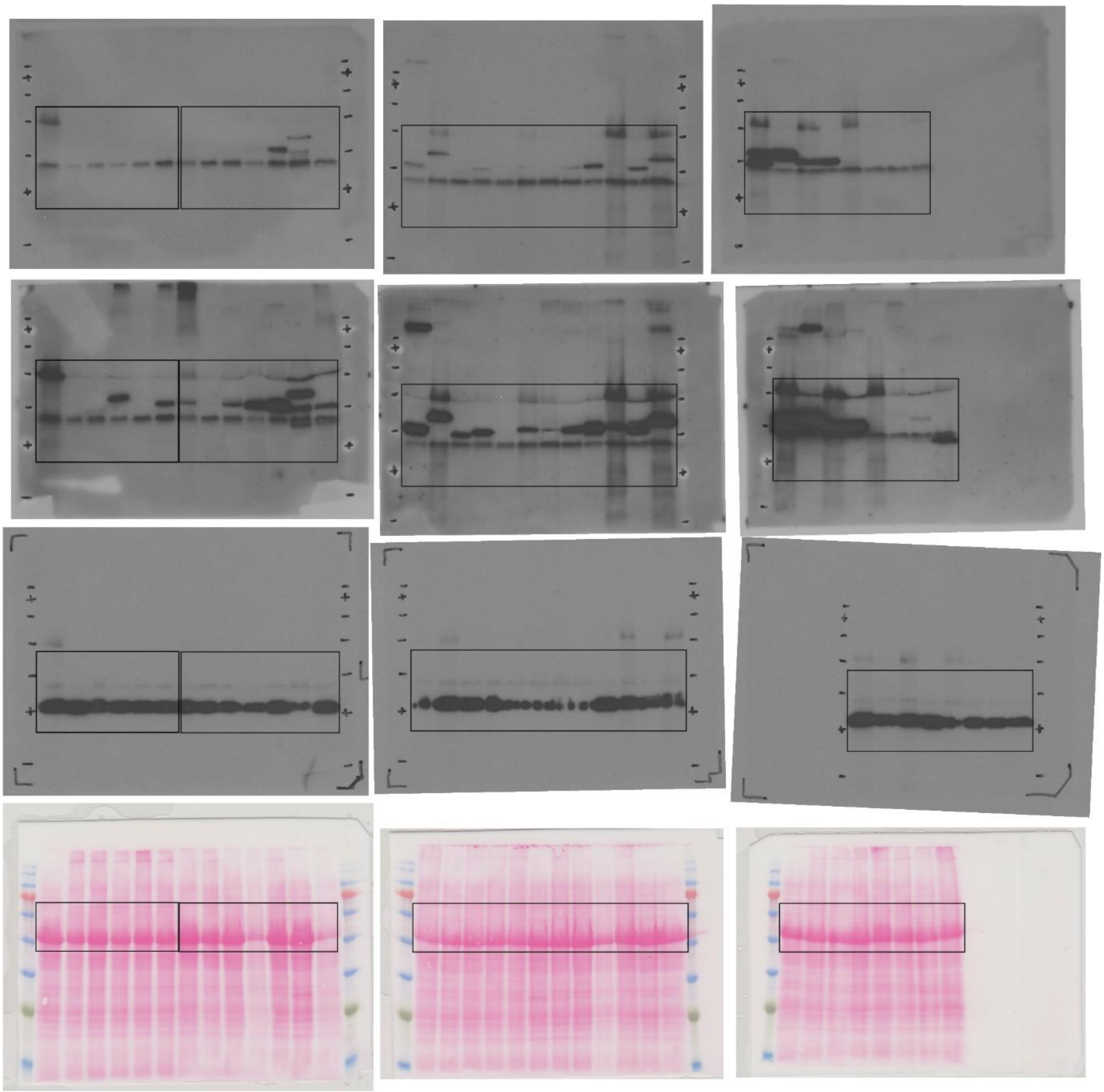

Supplementary Figure 12 Uncropped immunoblots and membranes shown in Supplementary Figure 1b.

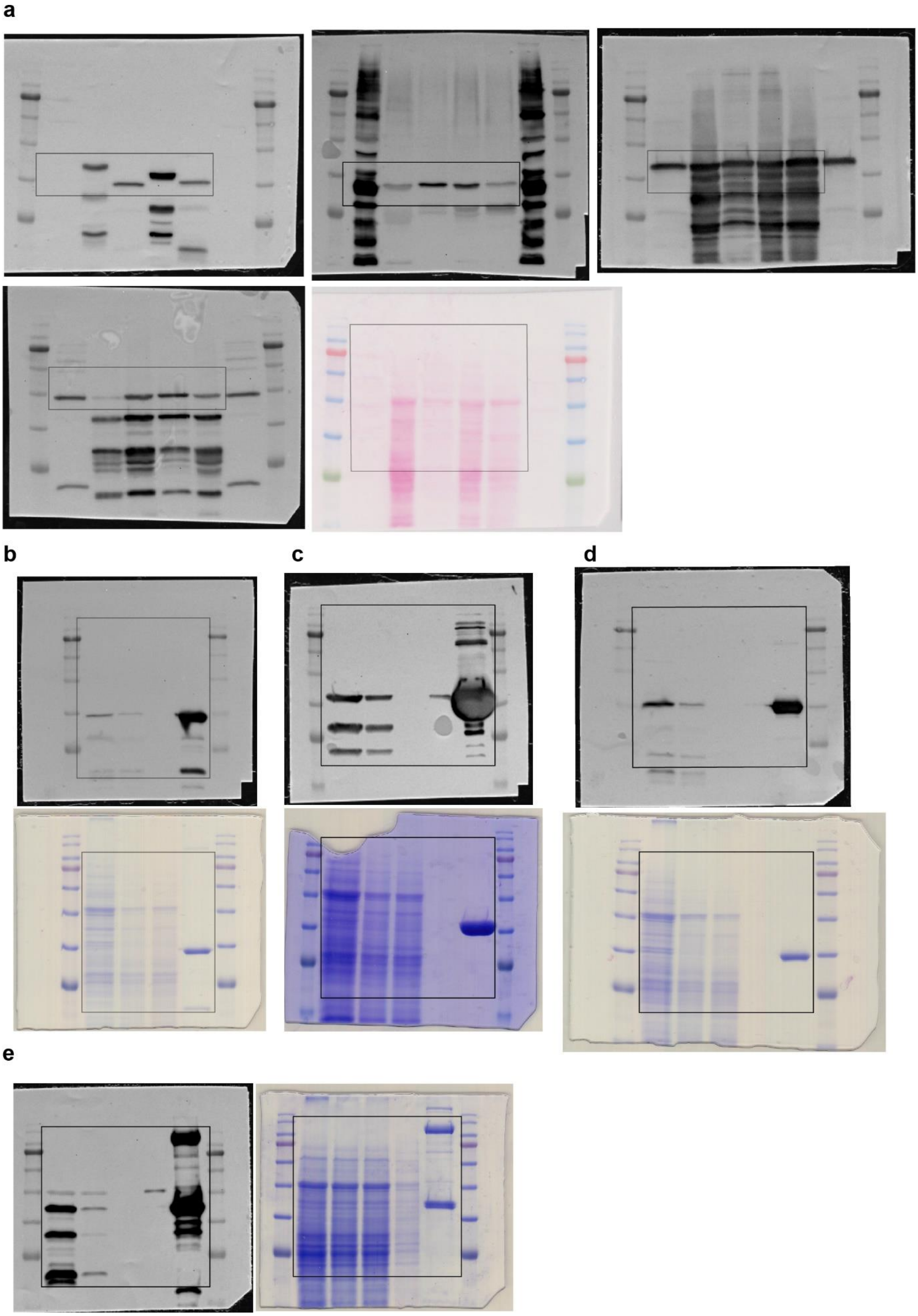

Supplementary Figure 13 Uncropped immunoblots, membrane and gels shown in Supplementary Figure 3. (**a**) Uncropped immunoblots and membrane shown in Supplementary Figure 3a. (**b**) Uncropped immunoblot and gel shown in Supplementary Figure

3b. (**c**) Uncropped immunoblot and gel shown in Supplementary Figure 3c. (**d**) Uncropped immunoblot and gel shown in Supplementary Figure 3d. (**e**) Uncropped immunoblot and gel shown in Supplementary Figure 3e.

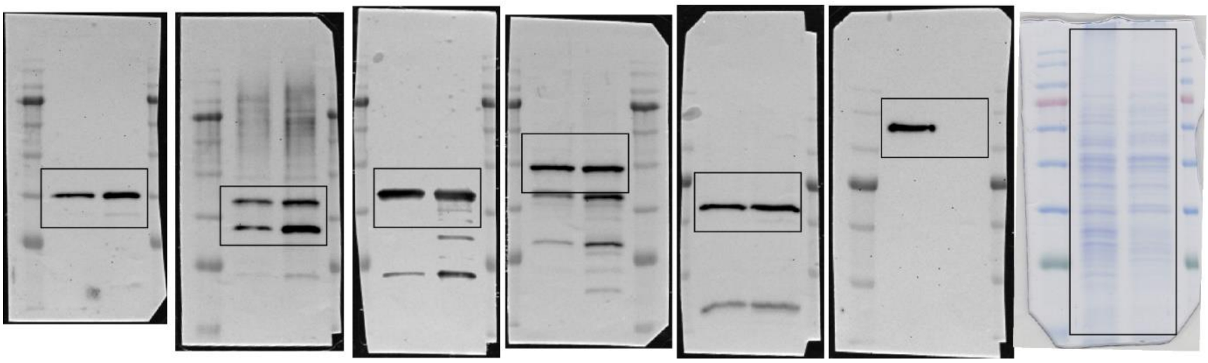

Supplementary Figure 14 Uncropped immunoblots and gel shown in Supplementary Figure 4.

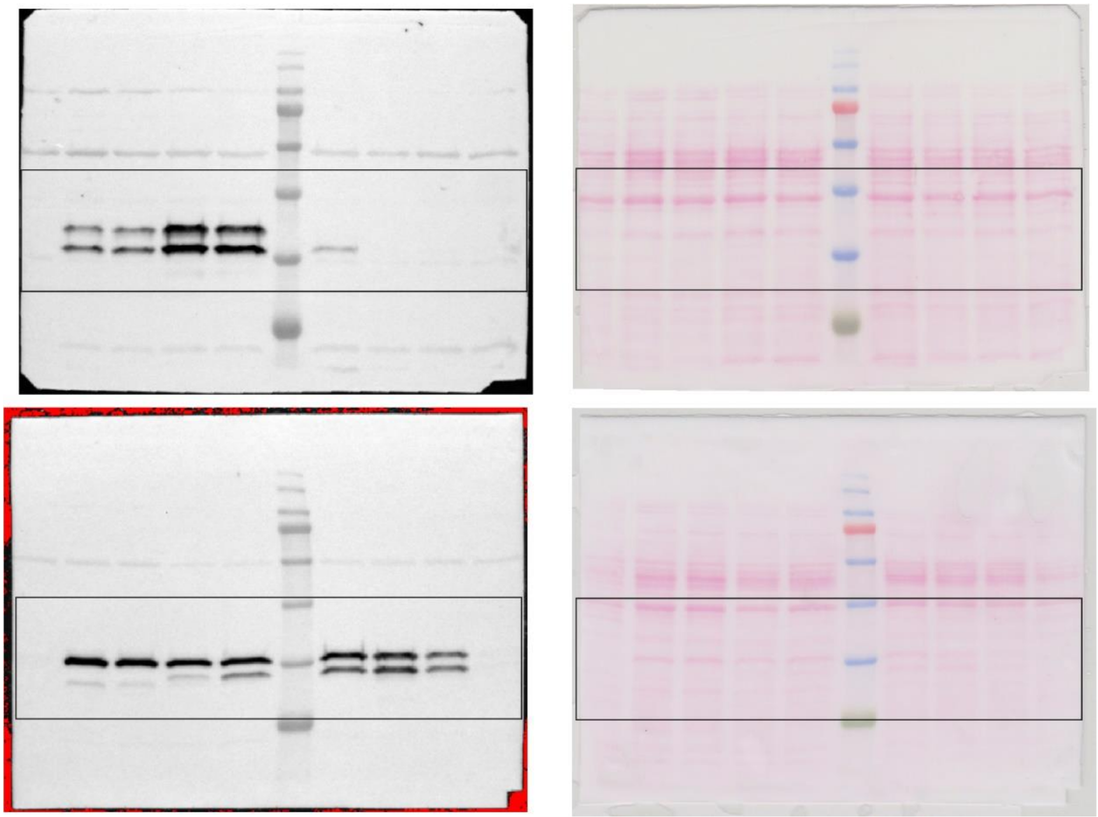

Supplementary Figure 15 Uncropped immunoblots and membranes shown in Supplementary Figure 6d.

a

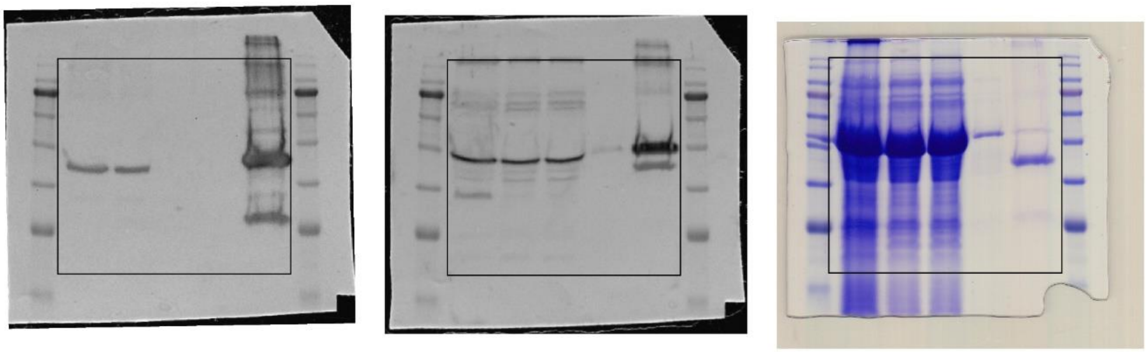

b

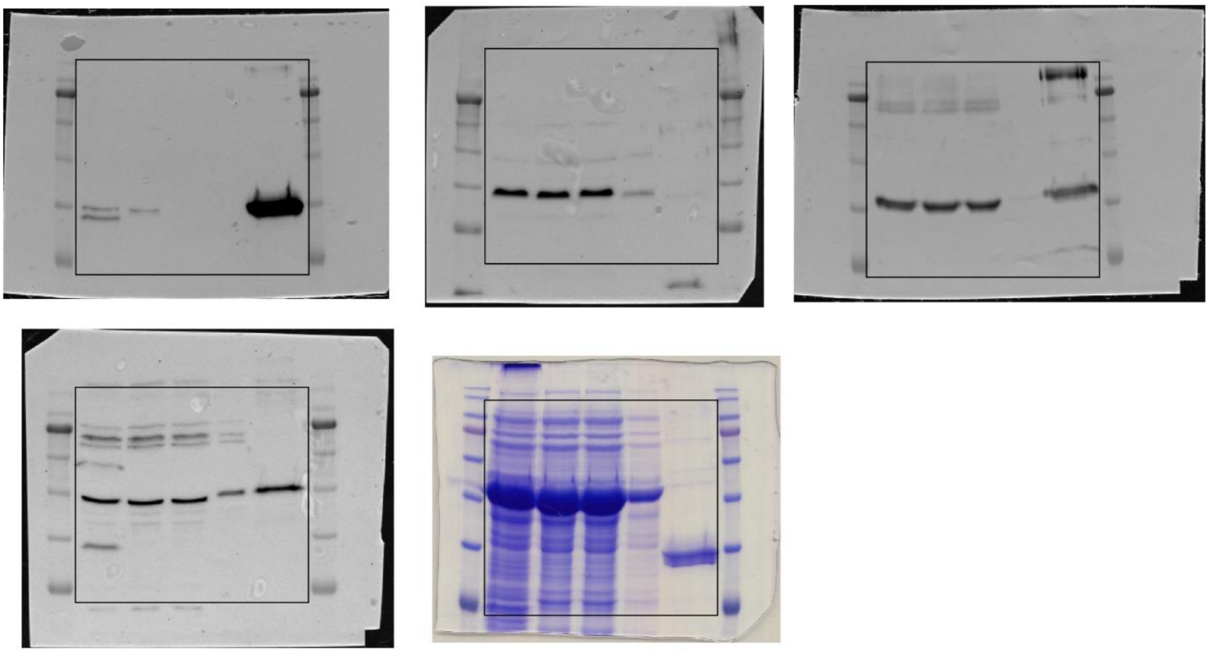

Supplementary Figure 16 Uncropped immunoblots and gels shown in Supplementary Figure 7. (**a**) Uncropped immunoblots and gel shown in Supplementary Figure 7b. (**b**) Uncropped immunoblots and gel shown in Supplementary Figure 7c.

Supplementary Tables

Supplementary Table 1 Plant vectors used in this study.

Plasmids	Expressed proteins	Promoter	Size (kDa)	
			FL	P
pN2XJ165	SU9-NifU^{Av}	p35S	40.8	33.6
	SU9-NifS^{Av}	p35S	51.3	44.0
	SU9-NifM^{Av}	pE35S	40.4	33.0
GB1203	p19	p35S	19.4	-
pN2XJ81	COX4-TS-NifH$^{\textit{Bradyrhizobium japonicum}}$	pE35S	34.7	38.2
	eGFP	p35S	27.0	-
pN2XJ82	COX4-TS-NifH$^{\textit{Rhizobium leguminosarum bv. trifolii WSM1689}}$	pE35S	38.3	34.8
	eGFP	p35S	27.0	-
pN2XJ83	COX4-TS-NifH$^{\textit{Azotobacter vinelandii (strain DJ)}}$	pE35S	38.2	34.7
	eGFP	p35S	27.0	-
pN2XJ84	COX4-TS-NifH$^{\textit{Herbaspirillum seropedicae (strain SmR1)}}$	pE35S	38.0	34.5
	eGFP	p35S	27.0	-
pN2XJ85	COX4-TS-NifH$^{\textit{Sinorhizobium meliloti (strain SM11)}}$	pE35S	38.6	35.2
	eGFP	p35S	27.0	-
pN2XJ86	COX4-TS-NifH$^{\textit{Nostoc sp. (strain PCC 7120)}}$	pE35S	39.4	36.0
	eGFP	p35S	27.0	-
pN2XJ87	COX4-TS-NifH$^{\textit{Gloeothece sp. KO68DGA}}$	pE35S	38.3	34.8
	eGFP	p35S	27.0	-
pN2XJ88	COX4-TS-NifH$^{\textit{Gluconacetobacter diazotrophicus (strain ATCC PAI5)}}$	pE35S	38.5	35.0
	eGFP	p35S	27.0	-
pN2XJ89	COX4-TS-NifH$^{\textit{Roseiflexus sp. (strain RS-1)}}$	pE35S	36.8	33.3
	eGFP	p35S	27.0	-
pN2XJ90	COX4-TS-NifH$^{\textit{Geobacter sulfurreducens (strain PCA)}}$	pE35S	38.1	34.6
	eGFP	p35S	27.0	-
pN2XJ91	COX4-TS-NifH$^{\textit{Pseudomonas stutzeri (strain DSM 4166)}}$	pE35S	38.4	34.9
	eGFP	p35S	27.0	-
pN2XJ92	COX4-TS-NifH$^{\textit{Nostoc sp. (strain PCC 6720)}}$	pE35S	38.9	35.4
	eGFP	p35S	27.0	-
pN2XJ93	COX4-TS-NifH$^{\textit{Ruminococcus albus SY3}}$	pE35S	38.0	34.5
	eGFP	p35S	27.0	-
pN2XJ94	COX4-TS-NifH$^{\textit{Paenibacillus sabinae T27}}$	pE35S	39.2	35.7
	eGFP	p35S	27.0	-
pN2XJ95	COX4-TS-NifH$^{\textit{Syntrophobacter fumaroxidans (strain DSM 10017)}}$	pE35S	36.4	32.9
	eGFP	p35S	27.0	-
pN2XJ96	COX4-TS-NifH$^{\textit{Clostridium pasteurianum BC1}}$	pE35S	36.4	32.9
	eGFP	p35S	27.0	-
pN2XJ97	COX4-TS-NifH$^{\textit{Rhodopseudomonas palustris}}$	pE35S	38.7	35.2
	eGFP	p35S	27.0	-
pN2XJ98	COX4-TS-NifH$^{\textit{Rhodobacter capsulatus (strain SB1003)}}$	pE35S	38.8	35.3
	eGFP	p35S	27.0	-
pN2XJ99	COX4-TS-NifH$^{\textit{Gloeothece citriformis (strain PCC 7424)}}$	pE35S	39.3	35.9

	eGFP	p35S	27.0	-
pN2XJ100	COX4-TS-NifH$^{\text{Nostoc azollae (strain 0708)}}$	pE35S	39.4	35.8
	eGFP	p35S	27.0	-
pN2XJ101	COX4-TS-NifH$^{\text{Synechococcus sp. (strain JA-2-3B'a(2-13))}}$	pE35S	38.6	35.1
	eGFP	p35S	27.0	-
pN2XJ102	COX4-TS-NifH$^{\text{Geobacter metallireducens (strain GS-15)}}$	pE35S	38.1	34.7
	eGFP	p35S	27.0	-
pN2XJ103	COX4-TS-NifH$^{\text{Azospirillum brasilense}}$	pE35S	38.3	34.9
	eGFP	p35S	27.0	-
pN2XJ104	COX4-TS-NifH$^{\text{Azoarcus sp. (strain BH72)}}$	pE35S	38.4	34.9
	eGFP	p35S	27.0	-
pN2XJ105	COX4-TS-NifH$^{\text{Methanothermobacter marburgensis (strain Marburg)}}$	pE35S	36.9	33.4
	eGFP	p35S	27.0	-
pN2XJ106	COX4-TS-NifH$^{\text{Methanocaldococcus infernus (strain ME)}}$	pE35S	37.7	34.3
	eGFP	p35S	27.0	-
pN2XJ107	COX4-TS-NifH$^{\text{Chlorobium tepidum (strain TLS)}}$	pE35S	36.8	33.3
	eGFP	p35S	27.0	-
pN2XJ108	COX4-TS-NifH$^{\text{Hydrogenobacter thermophilus (strain TK-6)}}$	pE35S	37.0	33.5
	eGFP	p35S	27.0	-
pN2XJ109	COX4-TS-NifH$^{\text{Methanothermobacter thermautotrophicus (strain Delta H)}}$	pE35S	39.7	36.2
	eGFP	p35S	27.0	-
pN2XJ110	COX4-TS-NifH$^{\text{Frankia sp. (strain FaC1)}}$	pE35S	38.1	34.6
	eGFP	p35S	27.0	-
pN2XJ111	COX4-TS-NifH$^{\text{Zymomonas mobilis subsp. mobilis (strain CP4)}}$	pE35S	38.7	35.2
	eGFP	p35S	27.0	-
pN2XJ112	COX4-TS-NifH$^{\text{Firmicutes bacterium CAG:536}}$	pE35S	33.8	30.3
	eGFP	p35S	27.0	-
pAE382	COX4-TS-NifH^{Ht}	2x CaMV p35S + 5'UTR TMV	37.0	33.5
	SU9-NifM^{Av}	p35S	40.4	33.0
	SU9-NifU^{Av}	p35S	40.8	33.6
	SU9-NifS^{Av}	p35S	51.3	44.0
	eGFP	p35S	27.0	-
	p19	p35S	19.4	-
pN2XJ198	COX4-TS-NifU^{Av}	pE35S	39.8	36.3
	SU9-NifS^{Av}	p35S	51.3	44.0
	p19	p35S	19.4	-

FL, full-length; P, processed by removal of mitochondria targeting peptide.

Supplementary Table 2 Yeast strains used in this study.

Strain	Plasmids	Expressed proteins	Promoter	Size (kDa)	
				FL	P
XJ1Y	pN2GLT4	SU9-NifU	GAL1	40.8	33.6
		SU9-NifS	GAL10	51.3	44.0
	pN2XJ187	SU9-NifM	GAL1	40.4	33.0
		COX4-TS-NifH^{Ht}	GAL10	37.0	33.5
XJ2Y	pN2GLT4	SU9-NifU	GAL1	40.8	33.6
		SU9-NifS	GAL10	51.3	44.0
	pN2XJ188	SU9-NifM	GAL1	40.4	33.0
		COX4-TS-NifH^{Mm}	GAL10	36.9	33.4
XJ3Y	pN2GLT4	SU9-NifU	GAL1	40.8	33.6
		SU9-NifS	GAL10	51.3	44.0
	pN2XJ189	SU9-NifM	GAL1	40.4	33.0
		COX4-TS-NifH^{Mi}	GAL10	37.7	34.3
XJ4Y	pN2GLT4	SU9-NifU	GAL1	40.8	33.6
		SU9-NifS	GAL10	51.3	44.0
	pN2XJ190	SU9-NifM	GAL1	40.4	33.0
		COX4-TS-NifH^{Av}	GAL10	38.2	34.7

FL, full-length; P, processed by removal of mitochondria targeting peptide.

Supplementary Table 3 *Sc*NifH^{Xx} purifications from yeast.

Strain	Protein	Yield (mg NifH per kg cells)	Fe per NifH dimer
XJ1Y	*Sc*NifH^{Ht}	227.0 ± 121.0	3.32 ± 1.78
XJ2Y	*Sc*NifH^{Mm}	28.6 ± 11.8	0.58 ± 0.26
XJ3Y	*Sc*NifH^{Mi}	199.0 ± 96.0	0.79 ± 0.04
XJ4Y	*Sc*NifH^{Av}	10.7 ± 6.7	1.41* ± 0.84

**Fe value not reliable due to different amounts of contaminants in different purifications.*

Supplementary Table 4 *Nb*NifU^{Av} purifications from tobacco.

Protein	Fe fertilization	Yield (mg NifU per kg leaves)	Fe per NifU dimer
*Nb*NifU^{Av}	YES	25.20 ± 6,22	2.24 ± 0.40
*Nb*NifU^{Av}	NO	8.541 ± 2.15	2.18 ± 0.40

Supplementary Table 5 *Nb*NifH^{Ht} purifications from tobacco.

***Nb*NifH^{Ht} purification**	Yield (mg NifH per kg leaves)	Fe per NifH dimer
1	7.20	bdl
2	3.76	bdl
3	3.63	bdl
4	5.35	bdl
5	8.32	bdl

bdl, below detection limit.

Supplementary References

1. Sarrion-Perdigones A*, et al.* GoldenBraid 2.0: a comprehensive DNA assembly framework for plant synthetic biology. *Plant Physiol* **162**, 1618-1631 (2013).

2. Buren S, Jiang X, Lopez-Torrejon G, Echavarri-Erasun C, Rubio LM. Purification and *In Vitro* Activity of Mitochondria Targeted Nitrogenase Cofactor Maturase NifB. *Front Plant Sci* **8**, 1567 (2017).

3. Page WJ, von Tigerstrom M. Optimal conditions for transformation of *Azotobacter vinelandii*. *J Bacteriol* **139**, 1058-1061 (1979).

4. Hernandez JA, Curatti L, Aznar CP, Perova Z, Britt RD, Rubio LM. Metal trafficking for nitrogen fixation: NifQ donates molybdenum to NifEN/NifH for the biosynthesis of the nitrogenase FeMo-cofactor. *Proc Natl Acad Sci U S A* **105**, 11679-11684 (2008).

5. Eseverri A, Lopez-Torrejon G, Jiang X, Buren S, Rubio LM, Caro E. Use of synthetic biology tools to optimize the production of active nitrogenase Fe protein in chloroplasts of tobacco leaf cells. *Plant Biotechnol J* **18**, 1882-1896 (2020).

6. Koskela EV, Frey AD. Homologous recombinatorial cloning without the creation of single-stranded ends: exonuclease and ligation-independent cloning (ELIC). *Mol Biotechnol* **57**, 233-240 (2015).

7. Naim F*, et al.* Advanced engineering of lipid metabolism in *Nicotiana benthamiana* using a draft genome and the V2 viral silencing-suppressor protein. *PLoS One* **7**, e52717 (2012).

8. Weber E, Engler C, Gruetzner R, Werner S, Marillonnet S. A modular cloning system for standardized assembly of multigene constructs. *PLoS One* **6**, e16765 (2011).

9. Werner S, Engler C, Weber E, Gruetzner R, Marillonnet S. Fast track assembly of multigene constructs using Golden Gate cloning and the MoClo system. *Bioeng Bugs* **3**, 38-43 (2012).

10. Waterhouse A*, et al.* SWISS-MODEL: homology modelling of protein structures and complexes. *Nucleic Acids Res* **46**, W296-W303 (2018).

11. Sievers F*, et al.* Fast, scalable generation of high-quality protein multiple sequence alignments using Clustal Omega. *Mol Syst Biol* **7**, 539 (2011).

12. Burgess BK, Lowe DJ. Mechanism of Molybdenum Nitrogenase. *Chem Rev* **96**, 2983-3012 (1996).

13. Altschul SF, Gish W, Miller W, Myers EW, Lipman DJ. Basic local alignment search tool. *J Mol Biol* **215**, 403-410 (1990).

14. Jacobson MR, Cash VL, Weiss MC, Laird NF, Newton WE, Dean DR. Biochemical and genetic analysis of the *nifUSVWZM* cluster from *Azotobacter vinelandii*. *Mol Gen Genet* **219**, 49-57 (1989).

15. Roberts GP, MacNeil T, MacNeil D, Brill WJ. Regulation and characterization of protein products coded by the *nif* (nitrogen fixation) genes of *Klebsiella pneumoniae*. *J Bacteriol* **136**, 267-279 (1978).
