# Supplementary material for: Exploiting genetic diversity and gene synthesis to identify superior nitrogenase NifH protein variants to engineer N2-fixation in plants
Source: Commun Biol. 2021 Jan 4;4:4. doi: 10.1038/s42003-020-01536-6 (PMC7782807; doi:10.1038/s42003-020-01536-6)
Supplement: Supplementary file 2 — Description of Additional Supplementary Files [file 42003_2020_1536_MOESM2_ESM.pdf]

Description of Additional Supplementary Files

File Name: Supplementary Data 1.
Description: Information about the NifH variants used in the library screening.

File Name: Supplementary Data 2.
Description: Raw data underlying the figure graphs.
